# Circadian gene variation in relation to breeding season and latitude in allochronic populations of two pelagic seabird species complexes

**DOI:** 10.1038/s41598-023-40702-8

**Published:** 2023-08-22

**Authors:** Katie Birchard, Hannah G. Driver, Dami Ademidun, Yuliana Bedolla-Guzmán, Tim Birt, Erin E. Chown, Petra Deane, Bronwyn A. S. Harkness, Austin Morrin, Juan F. Masello, Rebecca S. Taylor, Vicki L. Friesen

**Affiliations:** 1 Biology Department, Queen’s University, Kingston, ON K7L 3N6 Canada; 2 Grupo de Ecología y Conservación de Islas, 22800 Baja California, A.C Mexico; 3 Present Address: Apex Resource Management Solutions, Ottawa, ON K2A 3K2 Canada; 4 Present Address: Children’s Hospital of Eastern Ontario Research Institute, Ottawa, ON K1H 8L1 Canada; 5 Present Address: Mascoma LLC, Lallemand Inc., Lebanon, NH 03766 USA; 6 Present Address: Environment and Climate Change Canada, Wildlife Research Division, Ottawa, ON K1S 5B6 Canada; 7 Present Address: Sims Animal Hospital, Kingston, ON K7K 7E9 Canada; 8 Present Address: Department of Animal Behaviour, University of Bielefeld, 33615 Bielefeld, Germany; 9 Present Address: Environment and Climate Change Canada, Landscape Science and Technology Division, Ottawa, ON K1S 5R1 Canada

**Keywords:** Evolutionary biology, Population genetics, Ecology, Evolution, Genetics, Molecular biology, Ecology

## Abstract

Annual cues in the environment result in physiological changes that allow organisms to time reproduction during periods of optimal resource availability. Understanding how circadian rhythm genes sense these environmental cues and stimulate the appropriate physiological changes in response is important for determining the adaptability of species, especially in the advent of changing climate. A first step involves characterizing the environmental correlates of natural variation in these genes. Band-rumped and Leach’s storm-petrels (*Hydrobates* spp.) are pelagic seabirds that breed across a wide range of latitudes. Importantly, some populations have undergone allochronic divergence, in which sympatric populations use the same breeding sites at different times of year. We investigated the relationship between variation in key functional regions of four genes that play an integral role in the cellular clock mechanism—*Clock, Bmal1, Cry2* and *Per2*—with both breeding season and absolute latitude in these two species complexes. We discovered that allele frequencies in two genes, *Clock* and *Bmal1*, differed between seasonal populations in one archipelago, and also correlated with absolute latitude of breeding colonies. These results indicate that variation in these circadian rhythm genes may be involved in allochronic speciation, as well as adaptation to photoperiod at breeding locations.

## Introduction

### Importance of circadian rhythms

An organism’s fitness depends directly on its ability to synchronize important life history events with optimal resource availability^1^. In seasonal locations, annual cues in the environment result in physiological changes that allow organisms to time reproduction during periods of food abundance^2,3^. Photoperiod, or the number of hours of daylight during a twenty-four-hour period, varies based on time of year and latitude. Specifically, as latitude increases, photoperiod displays greater annual fluctuation. In some organisms, photoperiod has greater influence on breeding time than do other environmental cues such as temperature or precipitation^4,5^. Changes in photoperiod directly regulate circadian and circannual rhythms in many temperate species^6,7^. These rhythms modulate daily and annual changes in physiology and behaviour in response to environmental cues, and without them, organisms have reduced survival of offspring^3,8^.

**Figure 1:**
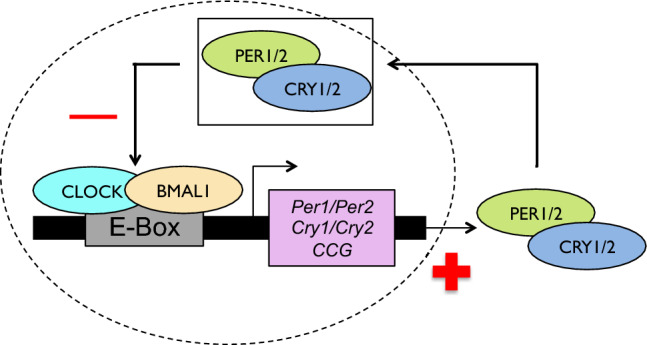
Core circadian gene mechanism. The dashed line represents the nuclear membrane. CLOCK and BMAL1 form a heterodimer in the cell nucleus in the presence of light, which binds to E-box regulatory elements in *Per* and *Cry* genes, as well as to other Clock Controlled Genes (CCG), causing transcription of PER and CRY (positive symbol). Once PER and CRY have accumulated to a threshold level, they form a heterodimer that enters the nucleus to repress CLOCK-BMAL1 (negative symbol). As PER and CRY degrade over time, CLOCK and BMAL1 can reform their complex, causing transcription of PER and CRY once again as the cycle repeats itself (positive symbol).

The clock machinery is highly complex, and multiple circadian rhythm genes are involved in sensing changes in photoperiod and stimulating appropriate physiological changes (e.g., for breeding). In this study we focus on *Clock, Per, Bmal1,* and *Cry*, as these genes transcribe protein products that form a significant part of the primary circadian clock mechanism in vertebrates^9,10^ (Table 1). The central role of these genes is to synchronize the internal circadian rhythm with external environmental cues^2,11^. The mechanism consists of positive and negative transcription/translation feedback loops that are expressed in many tissues, such as the suprachiasmatic nucleus of the anterior hypothalamus, mediobasal hypothalamus and pineal gland^11–13^ (Fig. 1). CLOCK and BMAL1 form a heterodimer in the cell nucleus in the presence of light, which binds to E-box regulatory elements in *Per* and *Cry* genes, activating transcription and translation into PER and CRY. Once PER and CRY have accumulated to a threshold level, they bind to casein kinase 1δ/ε to form a heterodimer, enter the nucleus, and mediate the displacement of CLOCK-BMAL1. As PER and CRY degrade over time, CLOCK and BMAL1 reform their complex and the cycle repeats itself. The CLOCK-BMAL1 heterodimer is involved in at least two additional regulatory loops promoting the transcription of other important circadian gene products (e.g., REV-ERBα and REV-ERBβ), which ultimately also affect the formation of CLOCK-BMAL1^14^. These feedback loops create rhythmic oscillations in gene expression that manifest as daily or seasonal changes in behaviour and physiology. A mutation in any of these genes can have pleiotropic effects, potentially causing molecular, physiological or behavioural arrhythmicity that may lead to reduced fitness [reviewed in^8,11,13^]. For example, mutations in several circadian genes reduce fertility in *Drosophila* [reviewed in 8]; and female blue tits (*Cyanistes caeruleus*) with fewer glutamine repeats fledge a higher number of offspring^15^.

**Table 1:** The core circadian clock genes.

Gene	Description
*Clock*	The *Clock* gene is the most well-studied circadian rhythm gene^10^. Studies involving knockout and mutant *Clock* genes in model species indicate a role for the gene in the rhythmic oscillation of other circadian-related genes and response to light cues^16,17^. The *Clock* gene sequence is largely conserved across taxa except for one region, which differs between individuals and species^18,19^. This region contains a variable number of trinucleotide repeats of CAA or CAG, both of which code for the amino acid glutamine (Q)^10^. This polyQ repeat region forms the transcription activation domain in the CLOCK protein. Interspecific differences in repeat number correspond with variation in transcription activity^20^. Thus, the role of the polyQ region on the molecular level affects circadian rhythms on the phenotypic level^20,21^
*Bmal1*	The transcription factor BMAL1 is likely involved in the onset of reproduction^22^. For example, adult birds have regressed gonads for most of the year^6,23^, but when photoperiodic change signals the onset of breeding time, BMAL1 stimulates the release of hormones to prepare the gonads for reproduction^24^. Mutations in *Bmal1* can lead to irregular levels of testosterone, luteinizing hormone, and follicle-stimulating hormone, as seen in *Bmal1* knockout mice^22^. While *Bmal1* is seemingly highly conserved across taxa, one study discovered that exon 15 differed between a nocturnal and diurnal bird species by a non-synonymous substitution in a casein kinase-1 regulatory site^25^. Thus, phosphorylation of *Bmal1* at this casein kinase-1 regulatory site may play a role in some behavioural phenotypes associated with circadian rhythm entrainment
*Per2*	In response to light detection, the *Per2* gene produces PER2, which is one of the transcription factors that represses the CLOCK-BMAL1 heterodimer^12,26–28^. Although research on functional variation in *Per2* is sparse, previous studies identified substitutions in exon 2 and exon 17 of this gene in humans^29,30^. Exon 17 of *Per2* codes for casein kinase-1 phosphorylation sites on PER2. Mutations in the casein kinase-1 phosphorylation sites in exon 17 can thus result in hypo-phosphorylation of PER2^30^. Phosphorylation of *Per2* may play a role in the degradation of PER2, and therefore hypo-phosphorylation could lead to abnormal behavioural responses to day and night cycles^30,31^
*Cry2*	*Cry2* has been relatively well-studied in humans, as mutations in this gene can cause sleep disorders such as Familial Advanced Sleep Phase^32^. In non-human vertebrates, effects of mutations in *Cry2* are less well-known, but the importance of flavine adenine dinuclueotide (FAD)-binding sites in cryptochromes has been documented in other taxa. For instance, in plants and *Drosophila*, FAD acts as a chromophore in light-sensitive proteins such as CRY2^33,34^. In response to light stimuli, FAD contributes to a signal transduction pathway that ultimately results in the stabilization of CRY2*.* When FAD binds to the CRY2 active site, it slows degradation and lengthens the circadian period^35^. Non-synonymous mutations in the amino acids of the FAD binding site (located in exons 6, 7, 8, and 9) could affect the ability of FAD to stabilize CRY2, and ultimately circadian entrainment

### Variation in circadian genes with breeding time and latitude

Circadian gene variation has been associated with differences in breeding time in both laboratory and wild populations. For example, in *Bmal1* knockout mice, irregular secretions of gonadotropin-releasing hormone resulted in smaller ovaries, smaller uteri, and delayed puberty^22,36,37^. Additionally, within wild populations of blue tits and Chinook salmon (*Oncorhynchus tshawytscha)*, individuals who bred earlier in the season had *Clock* alleles with shorter polyQ regions than individuals breeding later^15,38–40^. Variation in circadian gene allele frequencies have also been associated with latitude, suggesting that ecological parameters (e.g. photoperiod) that vary with latitude select for different alleles. For example, *Drosophila* exhibit a latitudinal cline in the number of Thr-Gly coding repeats in their *Per2* sequence^41,42^; and the cryptochrome gene is highly differentiated between high and low latitude *Drosophila* populations in Australia^43^. Although the relationship between latitude and individual genes from the core clock mechanism has been studied previously, the relationship between latitude and combined genes has not yet been defined.

### Storm-petrel species complexes as a study system

We explored the association between circadian gene allele variation with breeding season and latitude in the band-rumped and Leach’s storm-petrel species complexes (*Hydrobates* spp.; Procellariiformes: Hydrobatidae). Storm-petrels are small, pelagic seabirds that breed across a wide latitudinal range in both the North Atlantic and North Pacific oceans. These tube-nosed seabirds are generally philopatric, returning to their native breeding sites annually to reproduce. Multiple colonies have two sympatric allochronic populations, often breeding in the same nest sites in different seasons: one in winter and one in summer, with little to no overlap in breeding time. Seasonal populations have arisen independently within several archipelagoes, and represent a range of divergences from genetically similar populations, through genetically differentiated races, to reproductively isolated species in both complexes^44–47^. In this study we tested whether key functional regions of four core circadian genes vary (i) between populations with seasonal differences in breeding time, and/or (ii) between populations (including seasonal races and species) at different latitudes. Evidence from previous studies of natural populations of tits, salmon and D*rosophila* revealed that the number of glutamine repeats in the *Clock* gene correlates with both latitude and timing of breeding onset, possibly due to slower degradation of the longer glutamine tails (above). We therefore predicted that allele length would increase with breeding latitude in storm-petrels. We also predicted that *Clock* alleles would be longer in seasonal populations that initiate breeding when daylength is increasing (spring). Although the mechanisms of action of the other three genes are less well known in birds, we hypothesized that they also would vary by latitude and breeding season, changing phase, binding specificity or photo-entrainment as in other species.

## Methods

### Sample collection and preparation

**Figure 2:**
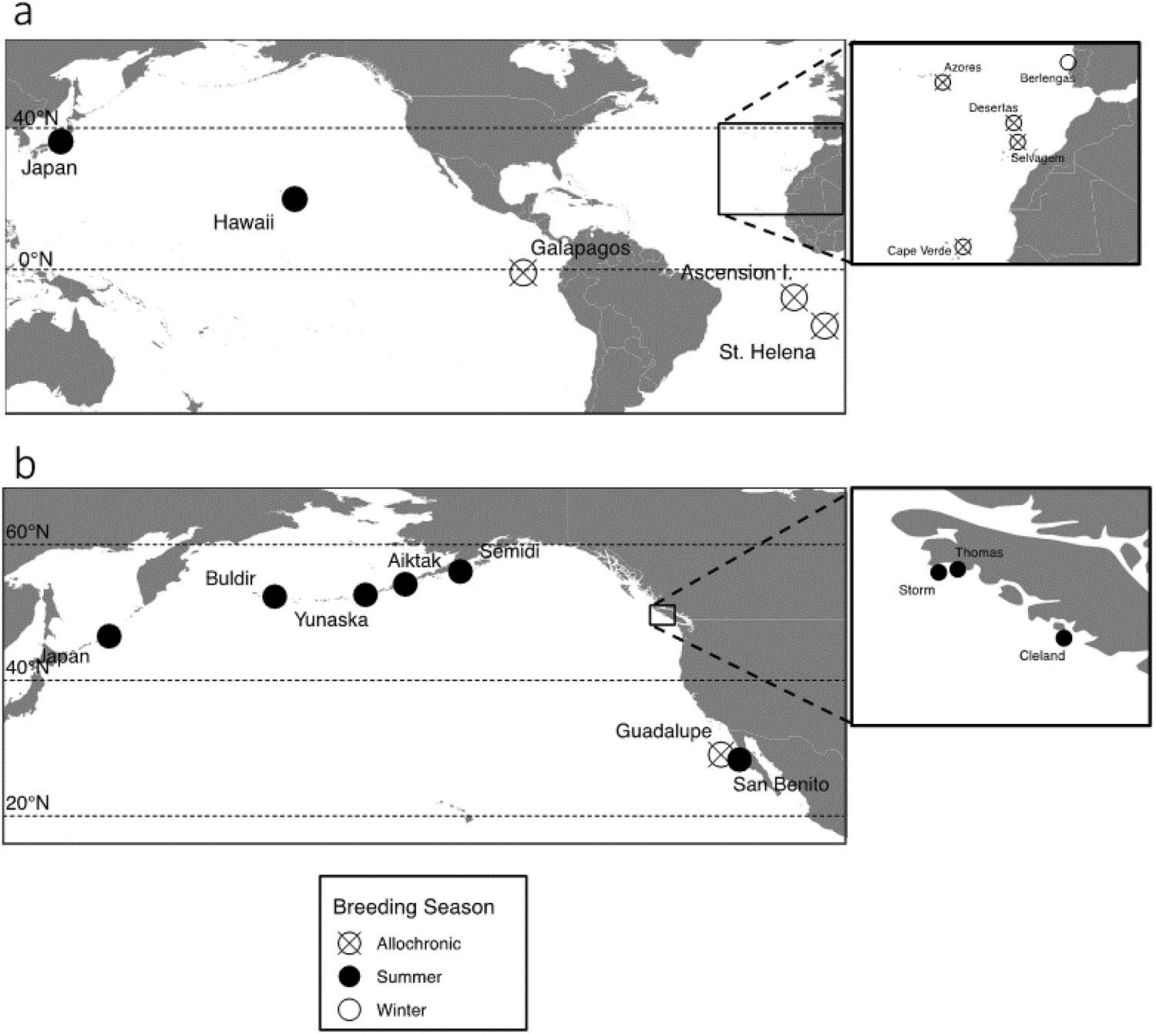
Sampling locations of (**a**) band-rumped, and (**b**) Leach’s storm-petrel species complexes (*Hydrobates* spp.). Filled circles indicate summer-breeding colonies, empty circles indicate winter-breeding colonies, and circles with a cross indicate allochronic populations. Boxes represent zoomed-in areas corresponding to plots to the right. The map was created in R using the *ggmap* package^50^.

Blood samples were collected from individual breeding band-rumped and Leach’s storm-petrels from throughout most of the species’ ranges, including seasonal populations (Fig. 2)^46,47^. All birds were handled under approved Animal Use Protocols, and were released immediately after sampling. The summer breeding season was classified as months experiencing increasing temperature and longer daylengths, and the winter breeding season as months experiencing decreasing temperature and shorter daylengths. Difficulties capturing birds mean that sample sizes at some colonies are small and variation may be underestimated. Blood samples were digested using proteinase-K, RNA contamination was removed using RNase, and DNA was extracted and cleaned using the phenol–chloroform method followed by ethanol precipitation^48^.

### Amplification and sequencing of circadian genes

Polymerase chain reaction (PCR) was performed for key functional portions of each of four core circadian clock genes. For *Clock*, forward (5'-TTT TCT CAA GGT CAG CAG CTT GT-3') and reverse (5'- CTG TAG GAA CTG TTG (C/T)GG (G/T)TG CTG-3') primers were previously designed to amplify a region of the gene with functional variation in polyQ repeats in avian species^16^. PCRs were performed in 10 μl reaction volumes, consisting of 5 μl Multiplex Mix (Qiagen, Mississauga, Ontario), 1.0 μl DNA, and 0.15 μl Dye 4—labeled M13 forward and reverse primers. A Biometra T-gradient Thermocycler (Biometrik Analytik, Goettingen, Germany) was used to conduct an initial denaturation at 95 °C for 15 min, followed by 35 cycles of 95 °C for 30 s, 55 °C for 45 s, and 72 °C for 45 s, and a final elongation phase of 72 °C for 3 min.

For *Bmal1*, forward (5’-CTA AAG TCA GCA TTT GAA AC-3’) and reverse (5’-CTC ACG TTG CTT GCC ACT AC-3’) primers were designed using the full genome of the Leach’s storm-petrel^51^ to amplify the 93 bp of exon 15. For *Per2*, each individual was genotyped for a 102 bp region corresponding to nucleotides 2536 to 2638 using previously designed forward (5’-TCT GGT AAA TCA AGT GGT CCY CCA GT-3’) and reverse (5’-TTC TAA TTC AGG TTG TGG CTT TTT GTC-3’) primers. Both these fragments were previously examined in barn owls (*Tyto alba*), chickens (*Gallus gallus*), and quails (*Coturnix japonica*), as these regions contain casein kinase 1e (CK1e) phosphorylation sites important for normal circadian function^25^. For *Cry2,* forward (5’-CCA GTC CTC CCC TGC TCG TG-3’) and reverse (5’-GAA ATG CCA GAC TCA CCC TG-3’) primers were designed from the Leach’s storm-petrel full genome to amplify 210 bp of exon 7, which is known to contain multiple flavin adenine dinucleotide (FAD)-binding sites^52^. PCRs were performed in 25 μl reaction volumes, consisting of 12.5 μl Multiplex Mix (Qiagen, Mississauga, Ontario), 0.04 μM forward primer, 0.04 μM reverse primer, and 2 μl DNA. PCRs were run as above but with annealing temperatures of 58 °C for *Bmal1*, 50 °C for *Per2*, and 60 °C for *Cry2*.

PCR products were electrophoresed through 2% agarose gels to confirm amplification. For *Clock*, 10 amplifications from Praia spring (Monteiro’s storm-petrel, *Hydrobates monteiroi*) and fall (band-rumped storm-petrel, *H. castro*) populations were sequenced by Genome Quebec (McGill University, Montreal, Quebec). The resulting sequences were aligned using BioEdit (version 7.0.9^53^) to confirm amplification of the intended target. A Beckman-Coulter CEQ 8000™ Genetic Analysis System (Core Genotyping Facility, Department of Biology, Queen’s University) was then used to screen for variation in allele lengths in all individuals. The Beckman-Coulter CE 8000 Genetic Analysis software (Beckman Coulter, Inc., Fullerton, CA) was used to score the resulting chromatograms. *Clock* allele lengths and sequences of 10 individuals were compared to determine the corresponding number of polyQ repeats based on allele length. Amplified products for all other genes were sequenced at Genome Quebec and aligned using Geneious^54^. Basic Local Alignment Search Tool (BLAST) searches on GenBank were used to confirm that the target genes were correctly amplified^55^. After base calls on the chromatograms were confirmed by eye, individual sequences were compared for variation.

Previously published data on microsatellite variation was used as a presumptively neutral variation to compare against variation in circadian genes^44,47,56^.

### Data analysis

To test for significant population differentiation within each gene or combination of genes, population genetic analyses were performed in ARLEQUIN^57^. Population classifications were determined by both breeding time and geographic location. First, *Bmal1* (total sample sizes (n): band-rumped storm-petrel n = 296, Leach’s storm-petrel n = 300)*, Cry2* (band-rumped storm-petrel n = 298, Leach’s storm-petrel n = 232)*,* and *Per2* (band-rumped storm-petrel n = 240, Leach’s storm-petrel n = 140) sequences were phased using the program DnaSP v5.10.1^58^ set to 1000 iterations and 100 burn-in. Then, ARLEQUIN was used to test genotypic data for deviations from Hardy–Weinberg proportions (HWP). ARLEQUIN was used to estimate Wright’s *F*_*ST*_, $\phi$_ST_ for *Bmal1, Cry2* and *Per2*, and Slatkin’s derivation of Wright’s *R*_*ST*_ for *Clock*^59^ between sampling sites and to test their statistical significance (10,100 permutations). Finally, ARLEQUIN was used to test for deviations from linkage equilibrium between pairs of circadian genes (10,100 permutations). Tests for deviations from linkage equilibrium were performed by grouping individuals from different colonies into genetic populations based on nonsignificant *F*_*ST*_ values and geographic proximity. For tests with greater than 20 comparisons, p-values were adjusted using the False Discovery Rate method and assessed for statistical significance using α = 0.05^60^. A Principal Components Analysis (PCA) was also performed on all samples that had sequences for all four genes (*Clock, Bmal1, Cry2,* and *Per2*) to help identify associations between haplotypes from different genes.

To test whether a significant relationship between circadian gene variation and breeding time or latitude exists, regressions were performed in R using the *stats* package^49^. For the *Bmal1* and *Cry2* sequences, two logistic regressions were run to determine whether the probability of having the most common allele can be predicted by latitude. Likelihood ratio tests and Akaike’s Information Criterion (AIC) of multiple generalized linear models were used to determine which variable(s) (ocean, species, breeding season and/or latitude), or interactions between variables, most accurately predicted presence/absence of specific alleles. Linear regressions were performed between latitude and *Clock* allele length, controlling for ocean in band-rumped storm-petrels. Due to non-normality of the residuals, confirmed by the Shapiro–Wilk test (band-rumped storm-petrels: W = 0.84, *p* < 0.001 n Atlantic = 724, n Pacific = 172; Leach’s storm-petrels: W = 0.81, *p* < 0.001, n = 366), estimates (intercept and slope) and measures of significance (p-values and 95% confidence intervals) of the model were obtained using 1000 bootstrap permutations (bootstrapped R^2^ values denoted with * in Results). Finally, the mean polyQ repeat number for each population was calculated and significant differences between allochronic populations were assessed using a Student’s t-test in R. While the use of mean allele length may obscure some features of allelic variation, it is deemed appropriate given the co-dominant nature of alleles at the *Clock* locus^44,61^.

Finally, to test whether significant correlations between circadian rhythm genes and latitude are better explained by geographic distance, Mantel tests^62^ were performed in R using the *ape* package. Genetic distances were estimated using Slatkin’s linearized pairwise *F*_*ST*_ values, and geographical distance was calculated as Euclidean distance between sampling sites based on latitude and longitude. Individuals from sympatric seasonal populations that were genetically indistinct based on Wright’s *F*_*ST*_ were grouped together for this analysis. For the band-rumped storm-petrels, samples from the Azores summer-breeding population and Cape Verde were removed from this analysis since they are considered separate species (Monteiro’s storm-petrel, and Cape Verde storm-petrel [*H. jabejabe*], respectively). Mantel tests were run using Pearson’s correlation for 10,000 permutations, controlling for either ocean (Atlantic or Pacific) or season (summer or winter). All tests were assessed for statistical significance using α = 0.05.

### Ethical approval

All samples were collected under Queen’s University University Animal Care Committee approved Animal Use Protocols, and birds were released immediately after handling (i.e. no experiments were conducted). All laboratory work was conducted in accordance with Queen’s University safety and biohazard regulations. All relevant Animal Research: Reporting of In Vivo Experiments (ARRIVE) guidelines were followed. Fieldwork was conducted under permits appropriate to each collection site. All methods were carried out in accordance with relevant guidelines and regulations.

## Results

### Circadian gene variation

Comparison with published avian sequences on GenBank confirmed that the focal fragments for all circadian genes were amplified correctly (see Table 2 for summaries of variation). *Per* was invariant across all samples for both species complexes. For *Clock,* all alleles differed by multiples of 3 bp and comparison of allele lengths and sequences indicated that variation in *Clock* alleles is due to the number of glutamine repeats.

**Table 2:** Circadian gene variation of band-rumped storm-petrels and Leach's storm-petrels. The sequence similarity indicates the percent similarity of the amplified sequence to the target sequence of GenBank.

Gene	Species	*n*	No. Pops	No. Alleles	Allele length (bp)	Sequence similarity (%)	Description
*Clock*	BSTP	382	18	7	277–298	98.2	*Clock* alleles comprised 6–13 glutamine repeats, in which *Clock* polyQ_11_ and *Clock* polyQ_12_, were the most prevalent, accounting for 26% and 53% of all alleles sampled, respectively. Only the Ascension summer population differed significantly from HWP with significantly lower heterozygosity than expected (* p* < 0.001; Supplementary Table S2)
	LESP	183	10	6	289–310		*Clock* alleles comprised 9–15 glutamine repeats, where *Clock* polyQ_11_ or *Clock* polyQ_12_ was the most common allele in every population, except in the Galapagos populations, where *Clock* polyQ_9_ was the most common. Only the San Benito population deviated significantly from HWP with a deficiency of heterozygotes (* p* < 0.001; Supplementary Tables S3, S4)
*Bmal1*	BSTP	154	15	5	93	95.7	Alleles 1 and 2 were the most prevalent, accounting for 88% and 8% of all alleles sampled. Observed heterozygosities within populations did not deviate from HWP
	LESP	150	10	6			Alleles 1 and 4 were the most prevalent, accounting for 76% and 17% of all alleles sampled. The Buldir population deviated from HWP with a deficiency of heterozygotes (Supplementary Tables S5–S8)
*Per2*	BSTP	120	11	1	102	97.4	No variable sites
	LESP	70	7	1			No variable sites
*Cry2*	BSTP	149	15	7	210	98.1	Alleles 1 and 2 were the most prevalent, accounting for 69% and 24% of all alleles sampled. Observed heterozygosities within populations did not deviate from HWP (Supplementary Tables S9–S12)
	LESP	116	10	15			Alleles 1 and 3 were the most prevalent, accounting for 53% and 30% of all alleles sampled. Observed heterozygosities within populations did not deviate from HWP (Supplementary Tables S9–S12)

BSTP = band-rumped storm-petrels, and LESP = Leach’s storm-petrels. HWP is Hardy–Weinberg Proportions.

Significant deviations from linkage equilibrium within genetic populations were found between *Bmal1* and *Clock* in band-rumped storm-petrels (average r^2^ = 0.00–0.05, *p* < 0.01), and between all pairs of genes tested in Leach’s storm-petrels (*Bmal1* ~ *Clock* average r^2^ = 0.00–0.06, *p* = 0.01, n = 276; *Cry2* ~ *Clock* average r^2^ = 0.00–0.07, *p* < 0.01, n = 216; *Cry2* ~ *Bmal1* average r^2^ = 0.00–0.22, *p* < 0.01). A PCA comparing clock gene haplotypes also indicated a potential correlation between *Cry2* and *Clock* variation in band-rumped storm-petrels, but not with the other clock genes and not in Leach’s storm-petrels (Supplementary Figure S1). Results from the linkage disequilibrium tests likely differed from the PCA for the Leach’s storm-petrels as the linkage disequilibrium tests looked for a relationship in each genetic population individually, whereas the PCA grouped all data together and only used samples that had been successfully sequenced for all four clock genes.

### Circadian gene variation and breeding season

Among allochronic band-rumped storm-petrel populations, only Azores summer and winter had estimates of *F*_*ST*_ significantly different from 0 based on *Clock* allele variation (*F*_*ST*_ = 0. 29, *p* < 0.01; Supplementary Table S14). Mean *Clock* polyQ lengths did not differ significantly between allochronic populations (t = 1.64, df = 893 *p* = 0.10). In allochronic populations of Leach’s storm-petrels on Guadalupe, pairwise estimates of* F*_*ST*_ did not differ from 0 (pairwise *F*_*ST*_ = 0.00, *p* = 0.57; Supplementary Tables S15, S16) and mean lengths of *Clock* alleles did not differ (t = 0.83, df = 71, *p* = 0.41).

Estimates of *F*_*ST*_ based on *Bmal1* allele frequencies did not differ from 0 between band-rumped storm-petrel or Leach’s storm-petrel populations separated by breeding time (Supplementary Tables S17–S20).

**Figure 3:**
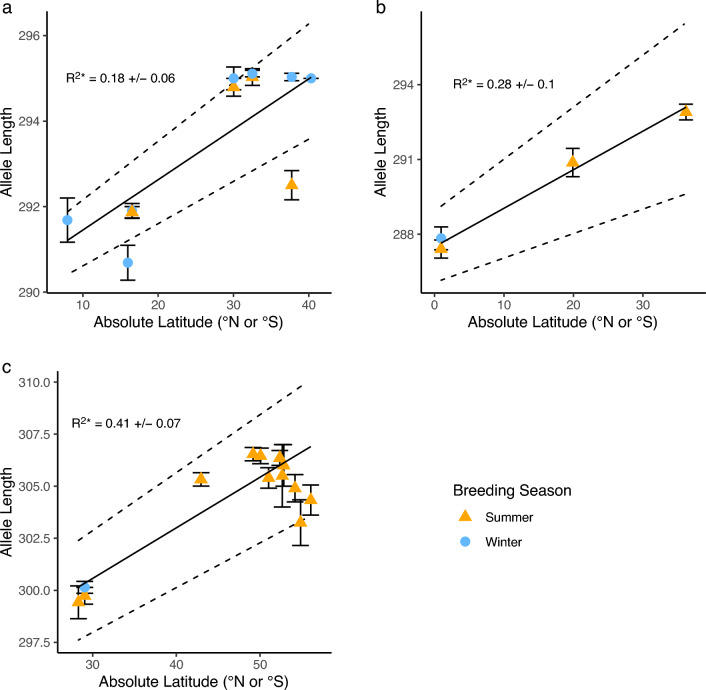
Relationship between *Clock* allele length and absolute latitude in (**a**) Atlantic band-rumped storm-petrels, (**b**) Pacific band-rumped storm-petrels, and (**c**) Leach’s storm-petrels. Points represent the average allele length for each population at a given absolute latitude and season, and vertical lines represent standard errors. Solid slanted lines represent the linear regression lines, while dashed slanted lines represent the 95% bootstrapped confidence intervals.

Pairwise estimates of *F*_*ST*_ for *Cry2* were significantly different from 0 between summer and winter breeding band-rumped storm-petrel populations in the Azores (*F*_*ST*_ = 0.49, *p* < 0.01). In the logistic regression model, breeding season was a significant predictor of *Cry2* alleles, with winter-breeding birds 3.17 times more likely to have allele 1 (*e*^βwinter^ = 3.17, 95% CI = 1.80–5.59, df = 2, n = 298, *p* < 0.01; Fig. 3). No other estimates of *F*_*ST*_ differed from 0 between seasonal populations in either species complex (all p > 0.05; Supplementary Tables S20–S24).

### Circadian gene variation and latitude

*Clock* allele length correlated significantly with absolute latitude (R^2^* = 0.18 ± 0.06 for Atlantic band-rumped storm-petrels, R^2^* = 0.28 ± 0.10 for Pacific band-rumped storm-petrels, and R^2^* = 0.41 ± 0.07 for Leach’s storm-petrels, all p* < 0.01, Fig. 3). Mantel tests also found a significant correlation between genetic and geographic distance in Atlantic band-rumped storm-petrel populations (r = 0.97, *p* = 0.03, 10,000 permutations). According to pairwise estimates of Slatkin’s linearized *F*_*ST*_, all higher latitude populations of Leach’s storm-petrels (Japan, Aleutians, Aiktak, British Colombia) except the Midun colony were significantly differentiated from lower latitude populations (San Benito, Guadalupe; *F*_*ST*_ = 0.52–3.38, all *p* < 0.01; Supplementary Table S15). A Mantel test for Leach’s storm-petrels found no significant correlation between genetic and geographic distance (r = 0.30, *p* = 0.09, 10,000 permutations).

**Figure 4:**
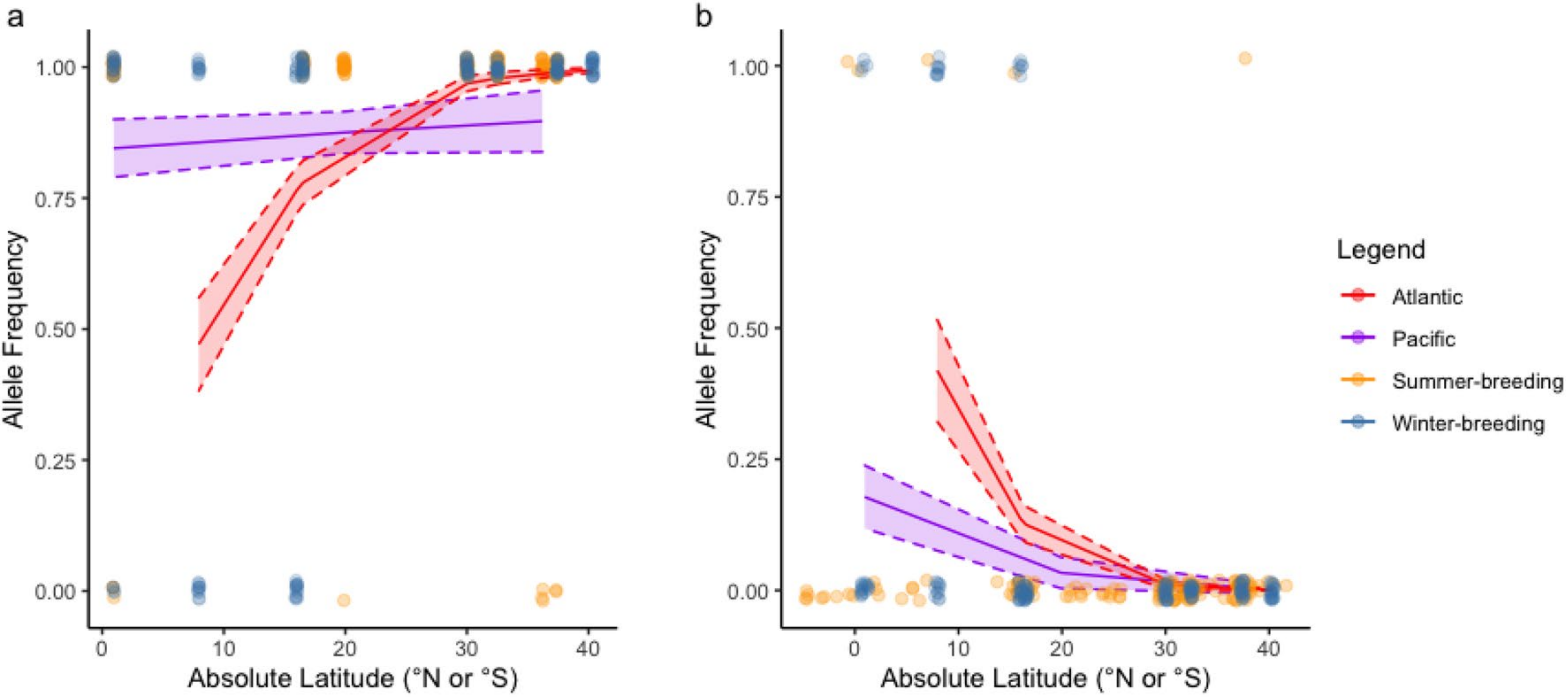
Relationship between frequency of *Bmal1* haplotypes and absolute latitude for (**a**) band-rumped storm-petrels, and (**b**) Leach’s storm-petrels. Each point represents whether an individual from a population has the most common allele for that species complex or not, and the colour of the point depicts whether that individual originates from a summer- or winter-breeding population. Solid lines represent the logistic regression curves for the most common haplotype in each species complex, controlling for ocean. Dashed lines represent the standard error surrounding each logistic regression curve.

Latitudinal variation was found in *Bmal1* for band-rumped storm-petrels*.* Based on two logistic regressions controlling for ocean, the probability of having allele 1 was significantly greater at higher absolute latitudes (*e*^β^ = 1.21, 95% CI = 1.11–1.31, df = 3, *p* < 0.01), whereas the probability of having allele 2 was significantly greater at lower latitudes (*e*^β^ = 0.80, 95% CI = 0.71 –0.90, df = 3, *p* < 0.01; Fig. 4; Supplementary Figure S2). The logistic regression was performed controlling for ocean because this was a significant predictor with latitude, according to likelihood ratio tests comparing all potential predictors. These results are consistent with the pairwise Slatkin’s linearized *F*_*ST*_ estimates indicating that the South Atlantic colonies have greater differentiation from colonies elsewhere (*F*_*ST*_ = 0.20–1.45, all *p* < 0.01; Supplementary Table S17). For Leach’s storm-petrels, the likelihood ratio test indicated that logistic regressions including absolute latitude as a predictor were not significantly better than the null model (χ^2^ = 4.29, df = 2, *p* = 0.12). Further, none of the pairwise estimates of Slatkin’s linearized *F*_*ST*_ for Leach’s storm-petrels were significant (Supplementary Tables S19, S20). No significant correlations were found between genetic and geographic distance using Mantel tests for either species complex (Atlantic band-rumped storm-petrels: r = 0.84, *p* = 0.07; Leach’s storm-petrels: r = -0.01, *p* = 0.44, 10,000 permutations each).

**Figure 5:**
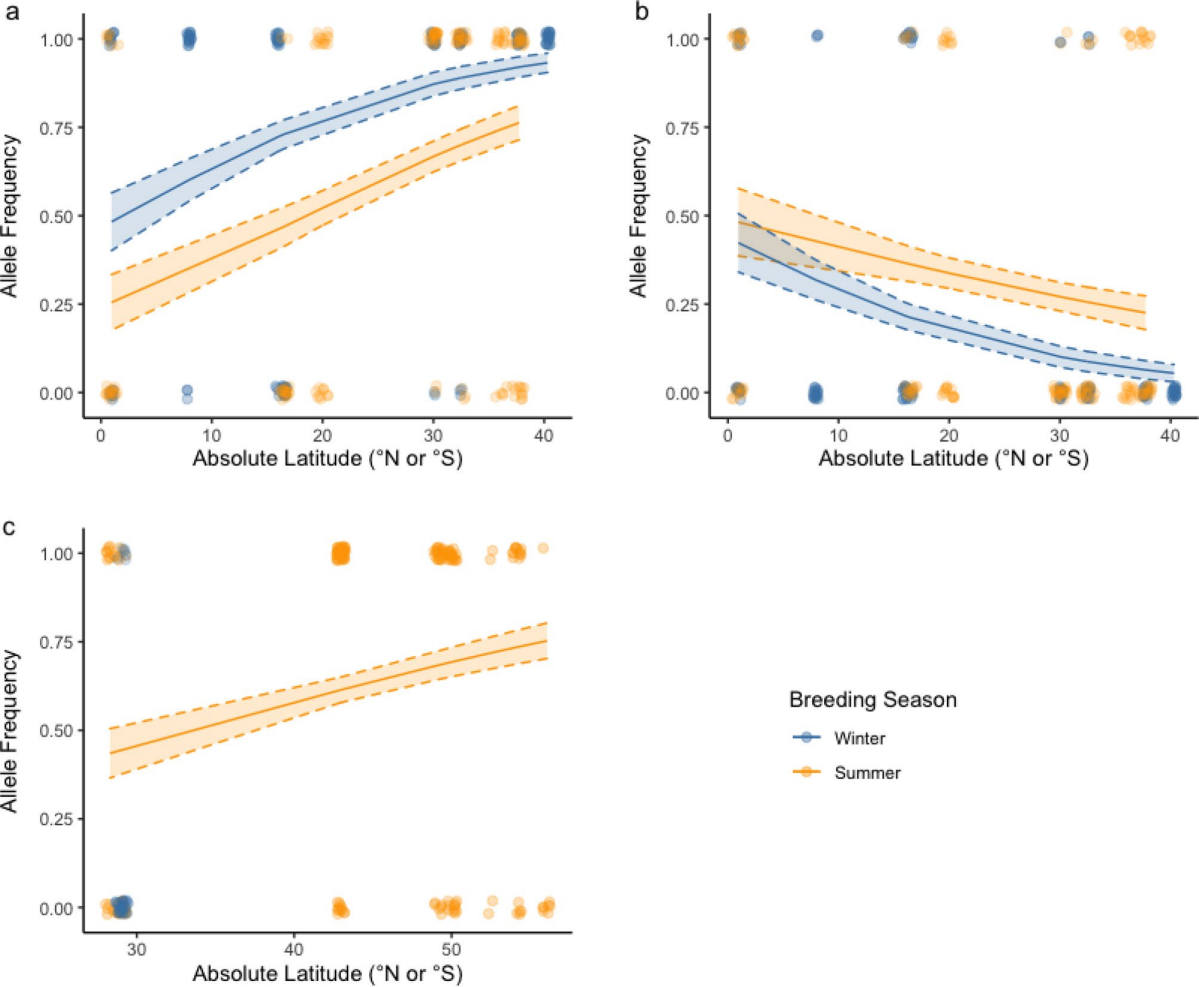
Relationship between frequency of *Cry2* haplotypes and absolute latitude for (**a**) allele 1 in band-rumped storm-petrels, (**b**) allele 2 in band-rumped storm-petrels and (**c**) allele 1 in Leach’s storm-petrels. Each point represents whether an individual from a population has the most common allele for that species complex or not, and the colour of the point depicts whether that individual originates from a summer- or winter-breeding population. Solid lines represent the logistic regression curves for the most common haplotype in each species complex, controlling for breeding season. Dashed lines represent the standard error surrounding each logistic regression curve.

Similar to *Clock* and *Bmal1*, *Cry2* variation also varied with latitude. When controlling for season, the frequency of allele 1 in band-rumped storm-petrels and Leach’s storm-petrels was significantly greater at higher absolute latitudes (band-rumped storm-petrels: *e*^β^ = 1.07, 95% CI = 1.04–1.09, df = 3, *p* < 0.01; Leach’s storm-petrels: *e*^β^ = 1.05, 95% CI = 1.02–1.09, df = 3, *p* < 0.01; Fig. 3; Supplementary Tables S4, S5). The frequency of allele 2 in band-rumped storm-petrels was significantly lower at higher absolute latitudes (band-rumped storm-petrels: *e*^β^ = 0.96, 95% CI = 0.94–0.98, df = 3, *p* < 0.01; Fig. 5). No significant correlations between genetic and geographic distance were found for either species complex from the Mantel’s tests (band-rumped storm-petrels: r = 0.53, *p* = 0.08; Leach’s storm-petrels: r = -0.03, *p* = 0.50, 10,000 permutations each).

## Discussion

In this study we aimed to characterize the relationship between variation in four core circadian rhythm genes and both breeding season and absolute latitude. To investigate this relationship, we explored variation in key functional regions in *Clock, Bmal1, Cry2* and *Per2* in two seabird species complexes containing populations that span a wide range of latitudes. Importantly, some of these populations have undergone allochronic divergence and even speciation within several archipelagos. We ultimately determined that circadian gene alleles (i) differ between seasonal populations within one but not all archipelagos, and (ii) vary among populations spanning different latitudes.

### Circadian gene variability in band-rumped and Leach’s storm-petrels

#### Variation in PolyQ length of Clock alleles

Although no new *Clock* alleles were detected in band-rumped or Leach’s storm-petrels compared to other species that have been studied, storm-petrels showed a greater total number of alleles at the *Clock* locus than do most other avian species studied to date, including the barn swallow (*Hirundo rustica*)^63,64^, bluethroat (*Luscinia svecica*)^38^, great tit (*Parus major*)^65^, and multiple species of tree swallow (*Tachycineta bicolor, T. thalassina, T. albilinae, T. leucorrhoa,* and *T. meyeni*)^66^.

The South Atlantic (St. Helena and Ascension Islands) populations of band-rumped storm-petrel and the San Benito Leach’s storm-petrel population deviated from HWP based on *Clock* allele frequencies. All these populations had heterozygote deficiencies, which can be due to a sampling artifact or population effects such as selection and non-random mating. In the case of the South Atlantic band-rumped storm-petrels, samples from two seasonal populations were grouped together (Supplementary Table S1), and thus the heterozygote deficiency could be due to the Wahlund effect, as neither population deviates from HWP when considered separately (Supplementary Table S2). San Benito comprises three small islands, and samples used in this analysis were taken from West, Central, and East Benito. Although the Wahlund effect is plausible for this group, neither a principal components analysis nor pairwise estimates of *F*_*ST*_ using SNPs from reduced representation sequencing detected genetic differentiation between islands (Supplementary Figure S3; Supplementary Table S25). In addition, none of six microsatellite loci analyzed in a previous study for Leach’s storm-petrels from San Benito deviated from HWP^47^, further indicating a lack of population substructure or inbreeding. Therefore, deviation of *Clock* genotypes from HWP in the San Benito population is more likely explained by purifying selection, selection against heterozygotes, or linkage disequilibrium (see below).

#### Variation in Bmal1

The two synonymous single nucleotide polymorphisms (SNPs) in *Bmal1* in band-rumped storm-petrels involve purine substitutions within adenine and guanine. Although synonymous, these mutations could influence the thermodynamic stability of the mRNA secondary structure, consequently affecting the speed of mRNA translation^67^. Degradation of BMAL1 is an important step in the negative feedback loop maintaining behavioural and physiological rhythms^27^, and a change in the speed of translation could alter the circadian phenotype.

In the Leach’s storm-petrels, the two non-synonymous mutations involved one heterozygous individual from Semidi Island with an aspartic acid in place of a glycine, and one homozygous individual from the Guadalupe winter-breeding population with a proline in place of a serine. Serine residues, unlike proline, are susceptible to phosphorylation^68^, thus the replacement of serine with proline results in the loss of potential phosphorylation sites. In mice, phosphorylation on serine 17 in BMAL1 results in decreased production of DNA binding protein, ultimately hindering transcription and reducing the efficacy of the circadian output^69^. If this mutation at nucleotide 73 in *Bmal1* exon 15 does indeed eliminate a phosphorylation site, then the Guadalupe individual may experience circadian arrhythmicity, consequently impacting its breeding phenology. Even though this mutation was only found in one of the sampled individuals, it may be prevalent in the population since the individual was homozygous at the site of the SNP (Supplementary Table S7).

#### Variation in Cry2

Despite the high diversity of *Cry2* alleles in band-rumped, and especially Leach’s storm-petrels (Table 2), only one mutation was non-synonymous, resulting in an amino acid change from isoleucine to phenylalanine. These two amino acids have similar biochemical properties and thus the mutation likely does not have a significant effect on protein function. The *Cry2* exon sequenced in this study centres on the FAD-binding site in the N-terminus, as this region is likely involved in blue light photo-sensing and therefore may be directly involved in entraining circadian rhythms^70,71^. The paucity of amino acid substitutions despite the high diversity of alleles is therefore not surprising given the conserved nature of the FAD-binding site and its important function^70^. Whether this region is highly variable in other avian species is uncertain, as the *Cry2* gene sequence is relatively unstudied in birds.

*Cry2* sequence variation has been studied extensively in mice, insects and plants, but findings from those studies may not provide much insight into *Cry2* function in the avian clock. For instance, many circadian genes are expressed differently across tissues in non-mammalian vertebrates^72^. In fact, Renthlei and colleagues^73^ discovered that CRY2 expression does not oscillate in the hypothalamus, pineal or intestine in tree sparrows. The pineal gland is thought to be vital to both circadian and circannual rhythms in some migratory songbirds^74–78^ (but see^79^) so the lack of a CRY2 oscillation may indicate that this gene plays a different role in the clock mechanism in birds compared to mammals. Ultimately, further research is required to explore fully the involvement of *Cry2* in the avian circadian clock mechanism and how its function differs from the mammalian circadian mechanism.

#### Variation in Per2

No variation in the *Per2* gene was detected across the 10 band-rumped storm-petrel populations or the seven Leach’s storm-petrel populations. The lack of variation is surprising given that both band-rumped and Leach’s storm-petrels show geographic variation at microsatellites, mitochondrial DNA, and other candidate genes^44,80,81^, and could indicate strong purifying selection on the *Per2* gene. Similarly, Fidler and Gwinner^25^ found that *Per2* was highly conserved among 12 day- and night-active avian species, and that the predicted translation products were identical.

#### Linkage disequilibrium

Linkage disequilibrium was found between *Clock* and *Bmal1* in band-rumped storm-petrels and between all combinations of *Bmal1*, *Clock*, and *Cry2* in Leach’s storm-petrels, indicating that particular allele combinations may offer a selective advantage. In addition, a PCA across all samples for allele variation in the three variable clock gene fragments in this study indicated a possible relationship between *Clock* and *Cry2* in the band-rumped storm-petrels (Supplementary Figure S1). Further, the alleles that correlate together for *Clock* and *Cry2* both tend to occur in higher latitudes.

The non-random association between *Clock* and *Bmal1* alleles for both storm-petrel species complexes is noteworthy, considering the relationship between the protein products of these two genes. Although the genes are located on different chromosomes^82,83^ and thus not physically linked, their protein products bind together to form the CLOCK-BMAL1 heterodimer, so the genes may have evolved together to preserve binding compatibility and/or enhance binding specificity. Research in mice determined that when CLOCK and BMAL1 bind together, the complex forms unique interfaces that interact with the *Per* and *Cry* genes, as well as with the PER-CRY heterodimer^84^. Mutations that affect these interfaces can lead to structural instability of the CLOCK-BMAL1 heterodimer, reduced transcriptional activity, and arrhythmic circadian oscillations^84^.

Photoperiod-related selection can also drive this pattern of linkage disequilibrium. All circadian genes in linkage disequilibrium show a latitudinal cline: *Clock* has a cline in the length of the polyQ tail, whereas *Cry2* and *Bmal1* have a cline in allele frequencies. However, the relative effects of environmental (e.g. photoperiod) and intrinsic (e.g. binding specificity) pressures are difficult to distinguish. To fully explore whether the binding specificity of protein products differs based on *Clock* allele length or *Bmal1* haplotype, and whether there is a relationship between binding specificity and latitude, further studies could compare levels of binding activity across latitudinally co-distributed variants^85^.

Finally, linkage disequilibrium can be the result of neutral processes, such as genetic drift. As population size decreases, combinations of alleles can arise due to random effects. Currently, Leach's storm-petrel populations are declining globally, and species breeding on Guadalupe Island are relatively small (2500–10,000 breeding pairs for both Townsend's *Hydrobates socorroensis* and Ainley's storm-petrels *H. cheimomnestes*)^86^. Although most band-rumped storm-petrel population sizes are large, populations that were recently elevated to species status in the Azores (Monteiro's storm-petrel) and Cape Verde (Cape Verde storm-petrel) are small (250–1000 and 15,500–67,500 breeding pairs respectively)^86^. These small population sizes suggest that *Bmal1* and *Clock* alleles may be inherited together due to chance, rather than because of environmental or intrinsic selection pressures.

### Comparison of circadian genes between allochronic populations

Allochronic populations of band-rumped and Leach’s storm-petrels provide an ideal opportunity to explore an association between circadian gene variation and breeding time. Neutral variation differs between summer and winter populations of band-rumped storm-petrels in the Azores and possibly Selvagem, and between seasonal races of Leach’s storm-petrels on Guadalupe Island^47,87^. In fact, the two seasonal populations of band-rumped storm-petrels in the Azores and Leach’s storm-petrels on Guadalupe Island are considered separate species^88^. Pairwise estimates of *F*_*ST*_ for both *Clock* and *Cry2* differed significantly between seasonal populations of band-rumped storm-petrels in the Azores, and breeding season was a significant predictor of *Cry2* alleles in the logistic regression model. Otherwise, no consistent patterns were detected based on breeding time. These results contrast with previous studies that found a correlation between the number of *Clock* polyQ repeats and breeding time in blue tits^15^ and barn swallows^64^. In both studies, fewer polyQ repeats were associated with earlier breeding times, and longer polyQ repeats were associated with later breeding times^15,64^. However, the conserved nature of the *Bmal1* exon indicates possible purifying selection between diverging lineages in both band-rumped and Leach’s storm-petrels, and may further highlight the importance of *Bmal1* functionality in regulating circadian rhythms.

The lack of a consistent pattern in circadian gene variation between seasonal breeding populations could be due to several factors. Firstly, evaluation of temporal patterns was based on population classification as either summer or winter breeders. This classification may have obscured other influences. For instance, seasonal populations may inhabit different non-breeding ranges and the photoperiod experienced during the non-breeding season may influence when individuals return to colonies to breed. Alternate classification criteria, such as population divergence time, may provide a better explanation for allelic differences between allochronic populations.

Secondly, lack of a consistent seasonal pattern in circadian gene variation may indicate that this variation does not influence breeding season. On a molecular level, determinants of breeding time in birds are complex and not fully understood^89^. In mammals and birds, photoperiod cues received in the retina signal the circadian clock to produce melatonin. In mammals, melatonin acts as a messenger to stimulate and regulate the gonadal axes^89^. In birds, however, melatonin production does not appear to stimulate gonads [^90^, but see^91^]. Additionally, other circadian genes could have a greater influence on breeding time than the gene fragments chosen in this study. For instance, avian *Cry4* is thought to be involved in the light-dependent magnetic compass for migration to the breeding grounds and, consequently, variation in this gene may correlate with breeding time^92^.

Thirdly, differences in circadian gene variation between allochronic populations may result from neutral processes, as supported by the relatively high divergence in neutral molecular markers between seasonal populations in the Azores. Furthermore, allochronic populations of storm-petrels tend to live close to the equator, where photoperiod is relatively constant; the only significant results were found at the archipelago farthest from the equator (the Azores). The lack of circadian gene variation between allochronic populations breeding at the same latitudes further supports the importance of latitude to circadian gene variation.

### Latitudinal variation in circadian genes

#### Latitudinal cline in polyQ length of Clock alleles

A significant latitudinal cline was detected in both band-rumped and Leach’s storm-petrels in *Clock* polyQ repeat number. Shorter polyQ repeat regions were more prevalent at lower latitudes, similar to non-migratory blue tits and salmon with variable spawning times^38–40^. The selective advantages of a longer or shorter polyQ tail are unknown, however within-populations, individuals with a greater number of polyQ repeats tend to display delayed phenology (e.g.,^93–95^). Although some studies have attributed differences in allele length to migratory phenotypes, as this would allow additional time to reach the breeding grounds before gonadal maturation, no consistent differences in *Clock* variation exist between migratory and non-migratory bird species^96^. For instance, although migratory bluethroats have significantly longer polyQ repeats at more southern latitudes^38^, an interspecies study comprising 23 trans-Saharan migratory bird species found that species breeding at more northern latitudes had significantly longer polyQ repeats^97^.

Further, glutamine trinucleotide repeats have a relatively high mutation rate compared to other microsatellites or SNPs, possibly facilitating rapid adaptation to different environmental pressures^98–100^. In fact, some evidence indicates that *Clock* proteins with shortened glutamine tails have weaker activation of *Per* and *Cry*, since the glutamine tail is important for binding to the E-box regulatory sequence that activates their transcription^101^. This difference in activation could lead to variation in the circadian period length across absolute latitudes^102^.

The lack of a significant correlation between geographic and genetic distance in microsatellites for the Leach’s storm-petrels^47^ supports the hypothesis that the latitudinal cline in *Clock* allele length in these populations is not due to geographic distance alone. In contrast, the significant correlation between geographic and genetic distance in Atlantic band-rumped storm-petrel populations indicates that distance-related genetic processes such as gene flow may be influencing polyQ repeat number, as colonies closer together share similar allele lengths. However, photoperiod also varies with geographic distance to the equator, and therefore could be positively autocorrelated with geographic distance between colonies. Spatial autocorrelation could result in a false conclusion that genetic drift is driving *Clock* allele length polymorphisms^103^.

#### Allelic diversity with latitude in Bmal1, Cry2, and Per2

The lack of diversity in *Bmal1* and *Cry2* at higher latitudes is surprising given the genetic structure of these two species complexes (Figs. 4 & 5). For instance, Cape Verde populations of band-rumped storm-petrels are considered a separate species^87,104^, yet these populations, in addition to other populations at higher absolute latitudes (Desertas, Selvagem, Berlengas, Japan, and Hawaii), are fixed for the most common *Bmal1* allele. Although more *Cry2* than *Bmal1* variants exist in samples from higher latitude populations, these populations also exhibit a significantly greater probability of having the *Cry2* allele 1. The presence of only a single *Bmal1,* and possibly *Cry2*, allele at higher latitudes in our sample could indicate the importance of their functions in regulating breeding time or adjusting the endogenous circadian rhythm to external cues when there is a greater fluctuation in photoperiod. In addition, since photoperiod is less variable at lower latitudes, and selection on the precision of the photoperiodic response is lower in more equatorial birds^25^, avian populations near the equator may hold greater levels of standing genetic variation.

However, it should be noted that within the Leach's storm-petrel species complex, species and absolute latitude co-vary, as one subspecies exists in higher latitude breeding populations, and the other species and subspecies exist closer to the equator. Thus, the pattern of allelic diversity in circadian genes in Leach's storm-petrels could also be explained by genetic differentiation between species and subspecies.

## Conclusion

This study has two main applications: a) understanding the relationship between *Clock*, *Bmal1*, *Per2*, and *Cry2* genes and circadian rhythms; and b) understanding mechanisms of allochronic speciation. Firstly, elucidating the relationship between *Clock* polyQ repeat number and differing *Bmal1* or *Cry2* variants in the avian clock orthologue is informative when analyzing genetic sequences in multiple species. We found some indications that genetic variation may correlate with breeding time, but stronger evidence that it may play a role in local adaptation to parameters correlated with latitude. Since only parts of the *Bmal1, Cry2*, and *Per2* genes were analyzed, future studies that capture the entire coding region of these genes and expand to other candidate circadian genes, such as *Cry1, Cry4* or *ADCYAP1*^104^, may provide better insight into how circadian gene variation correlates with latitude and/or breeding time.

Secondly, the Azores band-rumped storm-petrels and the Guadalupe Island Leach’s storm-petrels are some of the few documented examples of allochronic speciation in tetrapods^44,46^. Additionally, seasonal populations of band-rumped storm-petrels in Desertas, Selvagem, and the Galapagos are all possible examples of incipient speciation, each representing a different stage of speciation. The mechanisms of allochronic speciation, however, are not fully understood. Further research is required to fully understand the potential role of *Cry2* in facilitating allochronic speciation, possibly including more individuals from seasonal populations and a larger portion of the gene. The circadian clock mechanism is also highly complex, composed of other genes and regulatory loops that were not covered by this study. Future studies could focus on whether variation in these additional core clock genes is associated with allochronic speciation and whether these genes vary latitudinally. Finally, further research could explore mechanisms of action of these variants, circadian rhythm gene expression and epigenetic variation rather than sequences alone.

### Supplementary Information

Supplementary Information.

## Data Availability

The datasets used and/or analysed during the current study can be accessed through the Dryad data repository (https://doi.org/10.5061/dryad.dz08kps31) and GenBank (Accession No. OR223820-OR223859).
