# Supplementary material for: Circadian gene variation in relation to breeding season and latitude in allochronic populations of two pelagic seabird species complexes
Source: Sci Rep. 2023 Aug 22;13:13692. doi: 10.1038/s41598-023-40702-8 (PMC10444859; doi:10.1038/s41598-023-40702-8)
Supplement: Supplementary file 1 — Supplementary Information. [file 41598_2023_40702_MOESM1_ESM.docx]

**SUPPLEMENTARY MATERIAL**

**Circadian gene variation in relation to breeding season and latitude in allochronic populations of two pelagic seabird species complexes**

**Tables**

SUPPLEMENTARY TABLE S1. Number of gene copies (N), *Clock* allele frequencies for genetic populations of band-rumped storm-petrels, expected and observed heterozygosities (H_E_ and H_O_ respectively), and probabilities of deviation from Hardy-Weinberg proportions (p). Populations are arranged from north to south.

| **Allele** | **Japan** | **Hawaii** | **Galapagos** | **Azores Summer** | **North Atlantic** | **Cape Verde** | **South Atlantic** |
| --- | --- | --- | --- | --- | --- | --- | --- |
| N | 20 | 20 | 128 | 104 | 408 | 128 | 84 |
| 277 (polyQ_6_) | 0.00 | 0.00 | 0.01 | 0.00 | 0.00 | 0.00 | 0.00 |
| 283 (polyQ_8_) | 0.00 | 0.05 | 0.09 | 0.02 | 0.00 | 0.00 | 0.08 |
| 286 (polyQ_9_) | 0.00 | 0.00 | 0.56 | 0.14 | 0.01 | 0.03 | 0.00 |
| 289 (polyQ_10_) | 0.00 | 0.05 | 0.00 | 0.02 | 0.01 | 0.01 | 0.20 |
| 292 (polyQ_11_) | 0.70 | 0.90 | 0.34 | 0.28 | 0.01 | 0.93 | 0.56 |
| 295 (polyQ_12_) | 0.30 | 0.00 | 0.00 | 0.53 | 0.90 | 0.03 | 0.15 |
| 298 (polyQ_13_) | 0.00 | 0.00 | 0.00 | 0.01 | 0.07 | 0.00 | 0.00 |
| H_E_ | 0.44 | 0.19 | 0.57 | 0.63 | 0.19 | 0.13 | 0.62 |
| H_O_ | 0.40 | 0.20 | 0.53 | 0.63 | 0.18 | 0.14 | 0.29 |
| P | 1.00 | 1.00 | 0.49 | 0.62 | 0.41 | 1.00 | <0.001 |

SUPPLEMENTARY TABLE S2. Number of gene copies (N), *Clock* allele frequencies for all populations of the band-rumped storm-petrels, expected and observed heterozygosities (H_E_ and H_O_ respectively), and probabilities of deviation from Hardy-Weinberg proportions (p). Populations are arranged from north to south.

| **Allele** | **Japan** | **Hawaii** | **Galapagos Summer** | **Galapagos Winter** | **Berlengas** | **Azores Summer** | **Azores Winter** | **Desertas Summer** | **Desertas Winter** | **Selvagem Summer** | **Selvagem Winter** | **Cape Verde Summer** | **Cape Verde Winter** | **Ascension Summer** | **St. Helena Winter** |
| --- | --- | --- | --- | --- | --- | --- | --- | --- | --- | --- | --- | --- | --- | --- | --- |
| N | 20 | 20 | 66 | 62 | 26 | 104 | 92 | 96 | 54 | 72 | 68 | 52 | 76 | 36 | 48 |
| 277 (polyQ_6_) | 0.00 | 0.00 | 0.00 | 0.02 | 0.00 | 0.00 | 0.00 | 0.00 | 0.00 | 0.00 | 0.00 | 0.00 | 0.00 | 0.00 | 0.00 |
| 283 (polyQ_8_) | 0.00 | 0.05 | 0.08 | 0.11 | 0.00 | 0.02 | 0.00 | 0.01 | 0.00 | 0.00 | 0.00 | 0.00 | 0.00 | 0.08 | 0.08 |
| 286 (polyQ_9_) | 0.00 | 0.00 | 0.65 | 0.47 | 0.00 | 0.14 | 0.00 | 0.01 | 0.00 | 0.01 | 0.04 | 0.00 | 0.05 | 0.00 | 0.00 |
| 289 (polyQ_10_) | 0.00 | 0.05 | 0.00 | 0.00 | 0.00 | 0.02 | 0.00 | 0.01 | 0.00 | 0.04 | 0.00 | 0.00 | 0.01 | 0.14 | 0.25 |
| 292 (polyQ_11_) | 0.70 | 0.90 | 0.27 | 0.40 | 0.00 | 0.28 | 0.03 | 0.00 | 0.00 | 0.00 | 0.00 | 0.98 | 0.89 | 0.50 | 0.60 |
| 295 (polyQ_12_) | 0.30 | 0.00 | 0.00 | 0.00 | 1.00 | 0.53 | 0.92 | 0.86 | 0.96 | 0.89 | 0.82 | 0.02 | 0.04 | 0.28 | 0.06 |
| 298 (polyQ_13_) | 0.00 | 0.00 | 0.00 | 0.00 | 0.00 | 0.01 | 0.04 | 0.10 | 0.04 | 0.06 | 0.13 | 0.00 | 0.00 | 0.00 | 0.00 |
| H_E_ | 0.44 | 0.19 | 0.50 | 0.62 | 0.00 | 0.63 | 0.15 | 0.24 | 0.07 | 0.21 | 0.31 | 0.04 | 0.20 | 0.67 | 0.57 |
| H_O_ | 0.40 | 0.20 | 0.39 | 0.68 | 0.00 | 0.63 | 0.13 | 0.27 | 0.07 | 0.22 | 0.24 | 0.04 | 0.21 | 0.17 | 0.38 |
| p-value | 1.00 | 1.00 | 0.09 | 0.89 | 1.00 | 0.62 | 0.22 | 1.00 | 1.00 | 1.00 | 0.16 | 1.00 | 1.00 | <0.001 | 0.03 |

SUPPLEMENTARY TABLE S3. Number of gene copies (N), *Clock* allele frequencies for regional populations of Leach’s storm-petrels, expected and observed heterozygosities (H_E_ and H_O_ respectively), and probabilities of deviation from Hardy-Weinberg proportions (p). Populations are arranged from north to south.

| **Allele** | **Alaska Peninsula** | **Aleutian Islands** | **British Columbia** | **Japan** | **Guadalupe Summer** | **Guadalupe Winter** | **San Benito** |
| --- | --- | --- | --- | --- | --- | --- | --- |
| N | 20 | 64 | 78 | 70 | 38 | 56 | 40 |
| 289 (polyQ_9_) | 0.00 | 0.00 | 0.00 | 0.00 | 0.00 | 0.00 | 0.15 |
| 295 (polyQ_11_) | 0.00 | 0.00 | 0.00 | 0.00 | 0.21 | 0.14 | 0.05 |
| 301 (polyQ_13_) | 0.35 | 0.72 | 0.85 | 0.27 | 0.79 | 0.86 | 0.70 |
| 304 (polyQ_14_) | 0.00 | 0.00 | 0.00 | 0.01 | 0.00 | 0.00 | 0.05 |
| 307 (polyQ_15_) | 0.65 | 0.28 | 0.15 | 0.71 | 0.00 | 0.00 | 0.03 |
| 310 (polyQ_16_) | 0.00 | 0.00 | 0.00 | 0.00 | 0.00 | 0.00 | 0.03 |
| H_E_ | 0.48 | 0.41 | 0.26 | 0.42 | 0.34 | 0.25 | 0.49 |
| H_O_ | 0.30 | 0.38 | 0.26 | 0.46 | 0.32 | 0.14 | 0.25 |
| p-value | 0.48 | 0.67 | 1.00 | 1.00 | 1.00 | 0.07 | <0.001 |

SUPPLEMENTARY TABLE S4. Number of gene copies (N), *Clock* allele frequencies for all populations of Leach’s storm-petrels, expected and observed heterozygosities (H_E_ and H_O_ respectively), and probabilities of deviation from Hardy-Weinberg proportions (p). Populations are arranged from north to south.

| **Allele** | **Semidi** | **Midun** | **Aiktak** | **Chugi-danak** | **Yunaska** | **Buldir** | **Thomas** | **Storm** | **Cleland** | **Japan** | **Guadalupe Summer** | **Guadalupe Winter** | **San Benito** |
| --- | --- | --- | --- | --- | --- | --- | --- | --- | --- | --- | --- | --- | --- |
| N | 18 | 8 | 20 | 6 | 4 | 28 | 22 | 30 | 26 | 70 | 38 | 56 | 40 |
| 289 (polyQ_9_) | 0.00 | 0.00 | 0.00 | 0.00 | 0.00 | 0.00 | 0.00 | 0.00 | 0.00 | 0.00 | 0.00 | 0.00 | 0.15 |
| 295 (polyQ_11_) | 0.00 | 0.00 | 0.00 | 0.00 | 0.00 | 0.00 | 0.00 | 0.00 | 0.00 | 0.00 | 0.21 | 0.14 | 0.05 |
| 301 (polyQ_13_) | 0.44 | 0.63 | 0.35 | 0.17 | 0.25 | 0.11 | 0.09 | 0.27 | 0.08 | 0.27 | 0.79 | 0.86 | 0.70 |
| 304 (polyQ_14_) | 0.00 | 0.00 | 0.00 | 0.00 | 0.00 | 0.00 | 0.00 | 0.00 | 0.00 | 0.01 | 0.00 | 0.00 | 0.05 |
| 307 (polyQ_15_) | 0.56 | 0.38 | 0.65 | 0.83 | 0.75 | 0.89 | 0.91 | 0.73 | 0.92 | 0.71 | 0.00 | 0.00 | 0.03 |
| 310 (polyQ_16_) | 0.00 | 0.00 | 0.00 | 0.00 | 0.00 | 0.00 | 0.00 | 0.00 | 0.00 | 0.00 | 0.00 | 0.00 | 0.03 |
| H_E_ | 0.52 | 0.54 | 0.48 | 0.33 | 0.50 | 0.20 | 0.17 | 0.40 | 0.25 | 0.42 | 0.34 | 0.25 | 0.49 |
| H_O_ | 0.67 | 0.25 | 0.30 | 0.33 | 0.50 | 0.21 | 0.18 | 0.53 | 0.00 | 0.46 | 0.32 | 0.14 | 0.25 |
| p-value | 0.54 | 0.43 | 0.48 | 1.00 | 1.00 | 1.00 | 1.00 | 0.51 | 0.04 | 1.00 | 1.00 | 0.07 | <0.001 |

SUPPLEMENTARY TABLE S5. Number of gene copies (N), *Bmal1* frequencies for band-rumped storm-petrels, expected and observed heterozygosities (H_E_ and H_O_ respectively), and probabilities of deviation from Hardy-Weinberg proportions (p). Populations are arranged from north to south.

| **Allele** | **Japan** | **Hawaii** | | **Galapagos** | | **Azores Summer** | | **North Atlantic** | | **Cape Verde** | | | **South Atlantic** |
| --- | --- | --- | --- | --- | --- | --- | --- | --- | --- | --- | --- | --- | --- |
| N | 20 | | 22 | | 42 | | 22 | | 120 | | 42 | 40 | |
| 1 | 1.00 | | 0.95 | | 0.81 | | 0.91 | | 1.00 | | 1.00 | 0.40 | |
| 2 | 0.00 | | 0.05 | | 0.19 | | 0.05 | | 0.00 | | 0.00 | 0.35 | |
| 3 | 0.00 | | 0.00 | | 0.00 | | 0.05 | | 0.00 | | 0.00 | 0.03 | |
| 4 | 0.00 | | 0.00 | | 0.00 | | 0.00 | | 0.00 | | 0.00 | 0.15 | |
| 5 | 0.00 | | 0.00 | | 0.00 | | 0.00 | | 0.00 | | 0.00 | 0.08 | |
| H_E_ | 0.00 | | 0.09 | | 0.31 | | 0.17 | | 0.00 | | 0.00 | 0.69 | |
| H_O_ | 0.00 | | 0.09 | | 0.38 | | 0.18 | | 0.00 | | 0.00 | 0.65 | |
| p-value | 1.00 | | 1.00 | | 1.00 | | 1.00 | | 1.00 | | 1.00 | 0.22 | |

SUPPLEMENTARY TABLE S6. Number of gene copies (N), *Bmal1* allele frequencies for all populations of band-rumped storm-petrels, expected and observed heterozygosities (H_E_ and H_O_ respectively), and probabilities of deviation from Hardy-Weinberg proportions (p). Populations are arranged from north to south.

| **Allele** | **Japan** | **Hawaii** | **Galapagos Summer** | **Galapagos Winter** | **Berlengas** | **Azores Summer** | **Azores Winter** | **Desertas Summer** | **Desertas Winter** | **Selvagem Summer** | **Selvagem Winter** | **Cape Verde Summer** | **Cape Verde Winter** | **Ascension Summer** | **St. Helena Winter** |
| --- | --- | --- | --- | --- | --- | --- | --- | --- | --- | --- | --- | --- | --- | --- | --- |
|  |  |  |  |  |  |  |  |  |  |  |  |  |  |  |  |
| N | 20 | 20 | 22 | 20 | 20 | 22 | 20 | 20 | 18 | 22 | 20 | 26 | 18 | 20 | 20 |
| 1 | 0.85 | 0.95 | 0.82 | 0.80 | 1.00 | 0.91 | 1.00 | 1.00 | 1.00 | 1.00 | 1.00 | 1.00 | 1.00 | 0.50 | 0.30 |
| 2 | 0.00 | 0.05 | 0.18 | 0.20 | 0.00 | 0.05 | 0.00 | 0.00 | 0.00 | 0.00 | 0.00 | 0.00 | 0.00 | 0.40 | 0.30 |
| 3 | 0.15 | 0.00 | 0.00 | 0.00 | 0.00 | 0.05 | 0.00 | 0.00 | 0.00 | 0.00 | 0.00 | 0.00 | 0.00 | 0.00 | 0.05 |
| 4 | 0.00 | 0.00 | 0.00 | 0.00 | 0.00 | 0.00 | 0.00 | 0.00 | 0.00 | 0.00 | 0.00 | 0.00 | 0.00 | 0.10 | 0.20 |
| 5 | 0.00 | 0.00 | 0.00 | 0.00 | 0.00 | 0.00 | 0.00 | 0.00 | 0.00 | 0.00 | 0.00 | 0.00 | 0.00 | 0.00 | 0.15 |
| H_E_ | 0.27 | 0.10 | 0.31 | 0.34 | 0.00 | 0.18 | 0.00 | 0.00 | 0.00 | 0.00 | 0.00 | 0.00 | 0.00 | 0.61 | 0.79 |
| H_O_ | 0.30 | 0.10 | 0.36 | 0.40 | 0.00 | 0.18 | 0.00 | 0.00 | 0.00 | 0.00 | 0.00 | 0.00 | 0.00 | 0.50 | 0.80 |
| p-value | 1.00 | 1.00 | 1.00 | 1.00 | 1.00 | 1.00 | 1.00 | 1.00 | 1.00 | 1.00 | 1.00 | 1.00 | 1.00 | 0.77 | 0.19 |

SUPPLEMENTARY TABLE S7. Number of gene copies (N), *Bmal1* frequencies for Leach’s storm-petrels, expected and observed heterozygosities (H_E_ and H_O_ respectively), and probabilities of deviation from Hardy-Weinberg proportions (p). Populations are arranged from north to south.

| **Allele** | **Alaska Peninsula** | **Aleutian Islands** | **British Columbia** | **Japan** | **Guadalupe Summer** | **Guadalupe Winter** | **San Benito** |
| --- | --- | --- | --- | --- | --- | --- | --- |
| N | 20 | 40 | 62 | 64 | 34 | 46 | 34 |
| 1 | 0.90 | 0.78 | 0.79 | 0.75 | 0.79 | 0.78 | 0.56 |
| 2 | 0.00 | 0.00 | 0.00 | 0.00 | 0.00 | 0.04 | 0.00 |
| 3 | 0.00 | 0.03 | 0.00 | 0.00 | 0.00 | 0.07 | 0.00 |
| 4 | 0.10 | 0.18 | 0.19 | 0.22 | 0.15 | 0.04 | 0.26 |
| 5 | 0.00 | 0.03 | 0.02 | 0.03 | 0.06 | 0.04 | 0.18 |
| 6 | 0.00 | 0.00 | 0.00 | 0.00 | 0.00 | 0.02 | 0.00 |
| H_E_ | 0.19 | 0.38 | 0.34 | 0.39 | 0.35 | 0.39 | 0.60 |
| H_O_ | 0.20 | 0.15 | 0.42 | 0.38 | 0.41 | 0.30 | 0.76 |
| p | 1.00 | <0.01 | 0.65 | 0.08 | 1.00 | 0.10 | 0.16 |

SUPPLEMENTARY TABLE S8. Number of gene copies (N), *Bmal1* allele frequencies for all populations of Leach’s storm-petrels, expected and observed heterozygosities (H_E_ and H_O_ respectively), and probabilities of deviation from Hardy-Weinberg proportions (p). Populations are arranged from north to south. Midun, Chugidanak, and Yunaska were not included for this locus due to small sample sizes.

| **Allele** | **Semidi** | **Aiktak** | **Buldir** | **Thomas** | **Storm** | **Cleland** | **Japan** | **Guadalupe Summer** | **Guadalupe Winter** | **San Benito** |
| --- | --- | --- | --- | --- | --- | --- | --- | --- | --- | --- |
| N | 20 | 20 | 20 | 20 | 20 | 22 | 64 | 34 | 46 | 34 |
| 1 | 0.80 | 0.90 | 0.75 | 0.90 | 0.75 | 0.73 | 0.75 | 0.79 | 0.78 | 0.56 |
| 2 | 0.00 | 0.00 | 0.00 | 0.00 | 0.00 | 0.00 | 0.00 | 0.00 | 0.04 | 0.00 |
| 3 | 0.05 | 0.00 | 0.00 | 0.00 | 0.00 | 0.00 | 0.00 | 0.00 | 0.07 | 0.00 |
| 4 | 0.15 | 0.10 | 0.20 | 0.10 | 0.20 | 0.27 | 0.22 | 0.15 | 0.04 | 0.26 |
| 5 | 0.00 | 0.00 | 0.05 | 0.00 | 0.05 | 0.00 | 0.03 | 0.06 | 0.04 | 0.18 |
| 6 | 0.00 | 0.00 | 0.00 | 0.00 | 0.00 | 0.00 | 0.00 | 0.00 | 0.02 | 0.00 |
| H_E_ | 0.35 | 0.19 | 0.42 | 0.19 | 0.42 | 0.42 | 0.39 | 0.35 | 0.39 | 0.60 |
| H_O_ | 0.20 | 0.20 | 0.10 | 0.20 | 0.50 | 0.55 | 0.38 | 0.41 | 0.30 | 0.76 |
| p | 0.31 | 1.00 | 0.01 | 1.00 | 1.00 | 0.50 | 0.08 | 1.00 | 0.10 | 0.16 |

SUPPLEMENTARY TABLE S9. Number of gene copies (N), *Cry2* frequencies for band-rumped storm-petrels, expected and observed heterozygosities (H_E_ and H_O_ respectively), and probability of deviation from Hardy-Weinberg proportions (p). Populations are arranged from north to south.

| **Allele** | **Japan** | **Hawaii** | **Galapagos** | **Azores Summer** | **North Atlantic** | **Cape Verde** | **South Atlantic** |
| --- | --- | --- | --- | --- | --- | --- | --- |
| N | 18 | 22 | 40 | 26 | 124 | 30 | 38 |
| 1 | 0.72 | 0.59 | 0.43 | 0.58 | 0.92 | 0.07 | 0.82 |
| 2 | 0.28 | 0.41 | 0.48 | 0.38 | 0.08 | 0.40 | 0.18 |
| 3 | 0.00 | 0.00 | 0.03 | 0.04 | 0.00 | 0.00 | 0.00 |
| 4 | 0.00 | 0.00 | 0.00 | 0.00 | 0.00 | 0.47 | 0.00 |
| 5 | 0.00 | 0.00 | 0.00 | 0.00 | 0.00 | 0.07 | 0.00 |
| 6 | 0.00 | 0.00 | 0.05 | 0.00 | 0.00 | 0.00 | 0.00 |
| 7 | 0.00 | 0.00 | 0.03 | 0.00 | 0.00 | 0.00 | 0.00 |
| H_E_ | 0.42 | 0.51 | 0.61 | 0.54 | 0.15 | 0.63 | 0.31 |
| H_O_ | 0.56 | 0.27 | 0.60 | 0.38 | 0.13 | 0.13 | 0.26 |
| p | 1.00 | 0.21 | 0.56 | 0.25 | 0.32 | <0.001 | 0.49 |

SUPPLEMENTARY TABLE S10. Number of gene copies (N), *Cry2* allele frequencies for all populations of the band-rumped storm-petrels, expected and observed heterozygosities (H_E_ and H_O_ respectively), and probability of deviation from Hardy-Weinberg proportions (p). Populations are arranged from north to south.

| **Allele** | **Japan** | **Hawaii** | **Galapagos Summer** | **Galapagos Winter** | **Berlengas** | **Azores Summer** | **Azores Winter** | **Desertas Summer** | **Desertas Winter** | **Selvagem Summer** | **Selvagem Winter** | **Cape Verde Summer** | **Cape Verde Winter** | **Ascension Summer** | **St. Helena Winter** |
| --- | --- | --- | --- | --- | --- | --- | --- | --- | --- | --- | --- | --- | --- | --- | --- |
| N | 18 | 22 | 20 | 20 | 20 | 26 | 20 | 20 | 22 | 22 | 20 | 16 | 14 | 20 | 18 |
| 1 | 0.72 | 0.59 | 0.30 | 0.55 | 1.00 | 0.58 | 1.00 | 0.85 | 0.86 | 0.91 | 0.90 | 0.13 | 0.00 | 0.85 | 0.78 |
| 2 | 0.28 | 0.41 | 0.55 | 0.40 | 0.00 | 0.38 | 0.00 | 0.15 | 0.14 | 0.09 | 0.10 | 0.25 | 0.58 | 0.15 | 0.22 |
| 3 | 0.00 | 0.00 | 0.05 | 0.00 | 0.00 | 0.04 | 0.00 | 0.00 | 0.00 | 0.00 | 0.00 | 0.00 | 0.00 | 0.00 | 0.00 |
| 4 | 0.00 | 0.00 | 0.00 | 0.00 | 0.00 | 0.00 | 0.00 | 0.00 | 0.00 | 0.00 | 0.00 | 0.50 | 0.42 | 0.00 | 0.00 |
| 5 | 0.00 | 0.00 | 0.00 | 0.00 | 0.00 | 0.00 | 0.00 | 0.00 | 0.00 | 0.00 | 0.00 | 0.13 | 0.00 | 0.00 | 0.00 |
| 6 | 0.00 | 0.00 | 0.05 | 0.05 | 0.00 | 0.00 | 0.00 | 0.00 | 0.00 | 0.00 | 0.00 | 0.00 | 0.00 | 0.00 | 0.00 |
| 7 | 0.00 | 0.00 | 0.05 | 0.00 | 0.00 | 0.00 | 0.00 | 0.00 | 0.00 | 0.00 | 0.00 | 0.00 | 0.00 | 0.00 | 0.00 |
| H_E_ | 0.42 | 0.51 | 0.63 | 0.56 | 0.00 | 0.54 | 0.00 | 0.27 | 0.25 | 0.17 | 0.19 | 0.70 | 0.53 | 0.27 | 0.37 |
| H_O_ | 0.56 | 0.27 | 0.50 | 0.70 | 0.00 | 0.38 | 0.00 | 0.10 | 0.27 | 0.18 | 0.20 | 0.00 | 0.29 | 0.30 | 0.22 |
| p | 1.00 | 0.21 | 0.32 | 0.73 | 1.00 | 0.25 | 1.00 | 0.16 | 1.00 | 1.00 | 1.00 | <0.001 | 0.44 | 1.00 | 0.34 |

SUPPLEMENTARY TABLE S11. Number of gene copies (N), *Cry2* frequencies for Leach’s storm-petrels, expected and observed heterozygosities (H_E_ and H_O_ respectively), and probability of deviation from Hardy-Weinberg proportions (p). Populations are arranged from north to south.

| **Allele** | **Alaska Peninsula** | **Aleutian Islands** | **British Columbia** | **Japan** | **Guadalupe Summer** | **Guadalupe Winter** | **San Benito** |
| --- | --- | --- | --- | --- | --- | --- | --- |
| N | 18 | 12 | 58 | 58 | 30 | 40 | 16 |
| 1 | 0.72 | 0.25 | 0.71 | 0.79 | 0.17 | 0.15 | 0.63 |
| 2 | 0.06 | 0.00 | 0.03 | 0.02 | 0.00 | 0.00 | 0.13 |
| 3 | 0.22 | 0.33 | 0.26 | 0.19 | 0.53 | 0.43 | 0.13 |
| 4 | 0.00 | 0.00 | 0.00 | 0.00 | 0.03 | 0.03 | 0.00 |
| 5 | 0.00 | 0.00 | 0.00 | 0.00 | 0.17 | 0.20 | 0.00 |
| 6 | 0.00 | 0.00 | 0.00 | 0.00 | 0.00 | 0.03 | 0.00 |
| 7 | 0.00 | 0.00 | 0.00 | 0.00 | 0.00 | 0.05 | 0.00 |
| 8 | 0.00 | 0.00 | 0.00 | 0.00 | 0.00 | 0.05 | 0.00 |
| 9 | 0.00 | 0.00 | 0.00 | 0.00 | 0.00 | 0.05 | 0.00 |
| 10 | 0.00 | 0.00 | 0.00 | 0.00 | 0.03 | 0.03 | 0.00 |
| 11 | 0.00 | 0.00 | 0.00 | 0.00 | 0.00 | 0.00 | 0.13 |
| 12 | 0.00 | 0.00 | 0.00 | 0.00 | 0.07 | 0.00 | 0.00 |
| 13 | 0.00 | 0.25 | 0.00 | 0.00 | 0.00 | 0.00 | 0.00 |
| 14 | 0.00 | 0.08 | 0.00 | 0.00 | 0.00 | 0.00 | 0.00 |
| 15 | 0.00 | 0.08 | 0.00 | 0.00 | 0.00 | 0.00 | 0.00 |
| H_E_ | 0.45 | 0.82 | 0.44 | 0.34 | 0.68 | 0.76 | 0.60 |
| H_O_ | 0.33 | 0.83 | 0.41 | 0.28 | 0.80 | 0.80 | 0.75 |
| p-value | 0.53 | 0.71 | 0.81 | 0.40 | 0.67 | 0.40 | 1.00 |

SUPPLEMENTARY TABLE S12. Number of gene copies (N), *Cry2* allele frequencies for all populations of the Leach’s storm-petrels, expected and observed heterozygosities (H_E_ and H_O_ respectively), and probability of deviation from Hardy-Weinberg proportions (p). Populations are arranged from north to south.

| **Allele** | **Semidi** | **Aiktak** | **Buldir** | **Thomas** | **Storm** | **Cleland** | **Japan** | **Guadalupe Summer** | **Guadalupe Winter** | **San Benito** |
| --- | --- | --- | --- | --- | --- | --- | --- | --- | --- | --- |
| N | 8 | 18 | 4 | 18 | 14 | 26 | 58 | 30 | 40 | 16 |
| 1 | 0.13 | 0.72 | 0.50 | 0.56 | 0.79 | 0.77 | 0.79 | 0.17 | 0.15 | 0.63 |
| 2 | 0.00 | 0.06 | 0.00 | 0.06 | 0.00 | 0.04 | 0.02 | 0.00 | 0.00 | 0.13 |
| 3 | 0.25 | 0.22 | 0.50 | 0.39 | 0.21 | 0.19 | 0.19 | 0.53 | 0.43 | 0.13 |
| 4 | 0.00 | 0.00 | 0.00 | 0.00 | 0.00 | 0.00 | 0.00 | 0.03 | 0.03 | 0.00 |
| 5 | 0.00 | 0.00 | 0.00 | 0.00 | 0.00 | 0.00 | 0.00 | 0.17 | 0.20 | 0.00 |
| 6 | 0.00 | 0.00 | 0.00 | 0.00 | 0.00 | 0.00 | 0.00 | 0.00 | 0.03 | 0.00 |
| 7 | 0.00 | 0.00 | 0.00 | 0.00 | 0.00 | 0.00 | 0.00 | 0.00 | 0.05 | 0.00 |
| 8 | 0.00 | 0.00 | 0.00 | 0.00 | 0.00 | 0.00 | 0.00 | 0.00 | 0.05 | 0.00 |
| 9 | 0.00 | 0.00 | 0.00 | 0.00 | 0.00 | 0.00 | 0.00 | 0.00 | 0.05 | 0.00 |
| 10 | 0.00 | 0.00 | 0.00 | 0.00 | 0.00 | 0.00 | 0.00 | 0.03 | 0.03 | 0.00 |
| 11 | 0.00 | 0.00 | 0.00 | 0.00 | 0.00 | 0.00 | 0.00 | 0.00 | 0.00 | 0.13 |
| 12 | 0.00 | 0.00 | 0.00 | 0.00 | 0.00 | 0.00 | 0.00 | 0.07 | 0.00 | 0.00 |
| 13 | 0.38 | 0.00 | 0.00 | 0.00 | 0.00 | 0.00 | 0.00 | 0.00 | 0.00 | 0.00 |
| 14 | 0.13 | 0.00 | 0.00 | 0.00 | 0.00 | 0.00 | 0.00 | 0.00 | 0.00 | 0.00 |
| 15 | 0.13 | 0.00 | 0.00 | 0.00 | 0.00 | 0.00 | 0.00 | 0.00 | 0.00 | 0.00 |
| H_E_ | 0.86 | 0.45 | 0.67 | 0.57 | 0.36 | 0.38 | 0.34 | 0.68 | 0.77 | 0.60 |
| H_O_ | 0.75 | 0.33 | 1.00 | 0.56 | 0.14 | 0.46 | 0.28 | 0.80 | 0.80 | 0.75 |
| p-value | 0.66 | 0.53 | 1.00 | 0.72 | 0.23 | 1.00 | 0.40 | 0.68 | 0.48 | 1.00 |

SUPPLEMENTARY TABLE S13. Population pairwise estimates of Slatkin’s linearized *F_ST_* (below diagonal) and R_ST_ (above diagonal) based on variation in *Clock* alleles in band-rumped storm-petrels. Populations are arranged from north to south. N = North; S = South; (S) = summer-breeding. All p-values were corrected using the FDR method [37]. Statistical significance at p=0.05 and p=0.001 is indicated by * and **, respectively.

|  | **Japan** | **Hawaii** | **Galapagos** | **Azores (S)** | **N. Atlantic** | **Cape Verde** | **S. Atlantic** |
| --- | --- | --- | --- | --- | --- | --- | --- |
| **Japan** |  | 0.26* | 0.59** | -0.02 | 0.47** | 0.23* | 0.15* |
| **Hawaii** | 0.16* |  | 0.33** | 0.08* | 0.76** | 0.16* | -0.02 |
| **Galapagos** | 0.47** | 0.59** |  | 0.51** | 0.86** | 0.60** | 0.38** |
| **Azores (S)** | 0.18** | 0.59** | 0.38** |  | 0.42** | 0.02 | 0.07* |
| **N. Atlantic** | 2.06** | 4.20** | 2.19** | 0.41** |  | 0.69** | 0.68** |
| **Cape Verde** | 0.33** | 0.00 | 0.91** | 0.96** | 4.53** |  | 0.05 |
| **S. Atlantic** | 0.04 | 0.12* | 0.36** | 0.21** | 1.71** | 0.29** |  |

|  | **Japan** | **Hawaii** | **Gal (S)** | **Gal (W)** | **Berl** | **Az (S)** | **Az (W)** | **Des (S)** | **Des (W)** | **Sel (S)** | **Sel (W)** | **CV (S)** | **CV (W)** | **AI (S)** | **SH (W)** |
| --- | --- | --- | --- | --- | --- | --- | --- | --- | --- | --- | --- | --- | --- | --- | --- |
| **Japan** |  | 0.26* | 0.68** | 0.54** | 0.72** | -0.02 | 0.71** | 0.39** | 0.76** | 0.37** | 0.33* | 0.26* | 0.19* | 0.06 | 0.26* |
| **Hawaii** | 0.16 |  | 0.41** | 0.27** | 0.69** | 0.08* | 0.80** | 0.66** | 0.78** | 0.64** | 0.60** | 0.13* | 0.11* | 0.00 | -0.03 |
| **Gal (S)** | 0.69** | 0.91** |  | -0.01 | 0.82** | 0.55** | 0.88** | 0.84** | 0.86** | 0.82** | 0.81** | 0.67** | 0.65** | 0.49** | 0.38** |
| **Gal (W)** | 0.32** | 0.42** | 0.03 |  | 0.73** | 0.46** | 0.82** | 0.78** | 0.79** | 0.76** | 0.74** | 0.54** | 0.52** | 0.37** | 0.26** |
| **Berl** | 2.52** | 10.75** | 2.04** | 1.57** |  | 0.23** | -0.02 | -0.02 | 0.00 | -0.02 | -0.03 | 0.85** | 0.79** | 0.47** | 0.64** |
| **Az (S)** | 0.18* | 0.59** | 0.45** | 0.32** | 0.29** |  | 0.32** | 0.28** | 0.29** | 0.23** | 0.25** | 0.01 | 0.01 | 0.01 | 0.12* |
| **Az (W)** | 2.10** | 5.22** | 2.27** | 1.82** | 0.01 | 0.29** |  | -0.01 | -0.01 | 0.00 | -0.01 | 0.85** | 0.81** | 0.62** | 0.75** |
| **Des (S)** | 1.45** | 3.30** | 1.77** | 1.45** | 0.05 | 0.24** | 0.01 |  | -0.01 | 0.00 | -0.01 | 0.65** | 0.62** | 0.50** | 0.65** |
| **Des (W)** | 2.70** | 8.28** | 2.30** | 1.80** | 0.00 | 0.31** | 0.00 | 0.03 |  | 0.01 | -0.01 | 0.87** | 0.82** | 0.57** | 0.71** |
| **Sel (S)** | 1.60** | 3.89** | 1.82** | 1.47** | 0.03 | 0.24** | 0.00 | 0.00 | 0.01 |  | -0.01 | 0.66** | 0.62** | 0.46** | 0.62** |
| **Sel (W)** | 1.13** | 2.65** | 1.40** | 1.14** | 0.09* | 0.18** | 0.04* | 0.00 | 0.06* | 0.01 |  | 0.62** | 0.58** | 0.44** | 0.60** |
| **CV (S)** | 0.50* | 0.04 | 1.54** | 0.78** | 37.24** | 0.88** | 8.04** | 4.89** | 16.56** | 6.27** | 4.25** |  | -0.02 | -0.02 | 0.14* |
| **CV (W)** | 0.19* | 0.00 | 1.10** | 0.54** | 5.80** | 0.69** | 4.52** | 3.33** | 5.67** | 3.76** | 2.86** | 0.02 |  | -0.02 | 0.12* |
| **AI (S)** | 0.02 | 0.21* | 0.49** | 0.24** | 1.00** | 0.10* | 1.13** | 0.86** | 1.19** | 0.88** | 0.67** | 0.53** | 0.32** |  | 0.03 |
| **SH (W)** | 0.09* | 0.11* | 0.55** | 0.26** | 1.72** | 0.31** | 1.94** | 1.52** | 2.00** | 1.54** | 1.22** | 0.34** | 0.20** | 0.03 |  |

SUPPLEMENTARY TABLE S14. Population pairwise estimates of Slatkin’s linearized *F_ST_* (below diagonal) and R_ST_ (above diagonal) based on variation in *Clock* alleles in band-rumped storm-petrels. Populations are arranged from north to south. Az = Azores; Berl = Berlengas; SH = St. Helena; CV = Cape Verde; Des = Desertas; AI = Ascension Island; Gal = Galapagos; Sel = Selvagem; (S) = summer-breeding; (W) = winter-breeding. All p-values were corrected using the FDR method. Statistical significance at p=0.05 and p=0.001 is indicated by * and **, respectively.

SUPPLEMENTARY TABLE S15. Population pairwise estimates of Slatkin’s linearized *F_ST_* (below diagonal) and $\phi$_ST_ (above diagonal) based on variation in *Clock* alleles in Leach’s storm-petrels. Populations are arranged from north to south. Guad = Guadalupe; (S) = summer-breeding; (W) = winter-breeding. All p-values were corrected using the FDR method. Statistical significance at p=0.05 and p=0.001 is indicated by * and **, respectively.

|  | **Aiktak** | **Aleutians** | **BC** | **Japan** | **Guad (S)** | **Guad (W)** | **San Benito** |
| --- | --- | --- | --- | --- | --- | --- | --- |
| **Aiktak** |  | -0.02 | 0.09 | -0.02 | 0.65** | 0.67** | 0.42** |
| **Aleutians** | 0.00 |  | 0.03 | -0.02 | 0.69** | 0.69** | 0.55** |
| **BC** | 0.09 | 0.03 |  | 0.03 | 0.79** | 0.79** | 0.66** |
| **Japan** | 0.00 | 0.00 | 0.03 |  | 0.69** | 0.69** | 0.56** |
| **Guad (S)** | 0.81** | 1.04** | 2.00** | 1.02** |  | -0.01 | -0.02 |
| **Guad(W)** | 1.10** | 1.28** | 2.37** | 1.25** | 0.00 |  | 0.00 |
| **San Benito** | 0.52** | 0.75** | 1.45** | 0.75** | 0.05 | 0.06* |  |

SUPPLEMENTARY TABLE S16. Population pairwise estimates of Slatkin’s linearized *F_ST_* (below diagonal) and $\phi$_ST_ (above diagonal) based on variation in *Clock* alleles in Leach’s storm-petrels. Populations are arranged from north to south. Chugi = Chugidanak; Guad = Guadalupe; SB = San Benito; (S) = summer-breeding; (W) = winter-breeding. All p-values were corrected using the FDR method. Statistical significance at p=0.05 and p=0.001 is indicated by * and **, respectively.

|  | **Semidi** | **Midun** | **Aiktak** | **Chugi** | **Yunaska** | **Buldir-Agattu** | **Thomas** | **Storm** | **Cleland** | **Japan** | **Guad (S)** | **Guad (W)** | **SB** |
| --- | --- | --- | --- | --- | --- | --- | --- | --- | --- | --- | --- | --- | --- |
| **Semidi** |  | -0.03 | -0.04 | 0.05 | -0.09 | 0.23* | 0.25* | 0.03 | 0.29* | 0.03 | 0.59** | 0.60** | 0.36* |
| **Midun** | 0.00 |  | 0.06 | 0.24 | 0.07 | 0.48* | 0.50* | 0.18 | 0.55* | 0.18 | 0.46* | 0.47* | 0.20 |
| **Aiktak** | 0.00 | 0.07 |  | -0.03 | -0.15 | 0.13 | 0.14 | -0.03 | 0.18 | -0.02 | 0.65** | 0.67** | 0.42** |
| **Chugi** | 0.05 | 0.32 | 0.00 |  | -0.24 | -0.09 | -0.08 | -0.08 | -0.06 | -0.06 | 0.76** | 0.78** | 0.46* |
| **Yunaska** | 0.00 | 0.08 | 0.00 | 0.00 |  | -0.06 | -0.03 | -0.16 | 0.02 | -0.15 | 0.71* | 0.74** | 0.39* |
| **Buldir-Agattu** | 0.31* | 0.92* | 0.14 | 0.00 | 0.00 |  | -0.04 | 0.05 | -0.03 | 0.06 | 0.81** | 0.82** | 0.59** |
| **Thomas** | 0.33 | 1.00* | 0.16 | 0.00 | 0.00 | 0.00 |  | 0.06 | -0.04 | 0.07 | 0.82** | 0.83** | 0.58** |
| **Storm** | 0.03 | 0.22 | 0.00 | 0.00 | 0.00 | 0.05 | 0.06 |  | 0.08 | -0.02 | 0.70** | 0.71** | 0.50** |
| **Cleland** | 0.40* | 1.21* | 0.21 | 0.00 | 0.02 | 0.00 | 0.00 | 0.09 |  | 0.09 | 0.83** | 0.84** | 0.60** |
| **Japan** | 0.03 | 0.21 | 0.00 | 0.00 | 0.00 | 0.06 | 0.07 | 0.00 | 0.09 |  | 0.69** | 0.69** | 0.56** |
| **Guad (S)** | 0.55** | 0.21 | 0.81** | 1.56** | 1.13* | 2.29** | 2.39** | 1.13** | 2.63** | 1.02** |  | -0.01 | -0.02 |
| **Guad (W)** | 0.76** | 0.31 | 1.10** | 2.23** | 1.68* | 2.94** | 3.11** | 1.47** | 3.38** | 1.25** | 0.00 |  | 0.00 |
| **SB** | 0.33** | 0.08 | 0.52** | 0.92** | 0.63* | 1.49** | 1.51** | 0.76** | 1.67** | 0.75** | 0.05 | 0.06* |  |

SUPPLEMENTARY TABLE S17 Population pairwise estimates of Slatkin’s linearized *F_ST_* (below diagonal) and $\phi$_ST_ (above diagonal) based on variation in the *Bmal1* in band-rumped storm-petrels. Populations are arranged from north to south. All p-values were corrected using the FDR method. Significance at p=0.05 and p=0.001 is indicated by * and **, respectively.

|  | **Japan** | **Hawaii** | **Galapagos** | **Azores Hot** | **North Atlantic** | **Cape Verde** | **South Atlantic** |
| --- | --- | --- | --- | --- | --- | --- | --- |
| **Japan** |  | 0.00 | 0.00 | 0.00 | 0.00 | 0.00 | 0.67* |
| **Hawaii** | 0.00 |  | 0.00 | 0.00 | 0.00 | 0.00 | 0.64* |
| **Galapagos** | 0.13 | 0.04 |  | 0.00 | 0.00 | 0.00 | 0.58* |
| **Azores Hot** | 0.02 | 0.00 | 0.03 |  | 0.00 | 0.00 | 0.65* |
| **North Atlantic** | 0.00 | 0.15 | 0.43** | 0.21 |  | 0.00 | 0.85** |
| **Cape Verde** | 0.00 | 0.05 | 0.21* | 0.07 | 0.00 |  | 0.75** |
| **South Atlantic** | 0.50** | 0.38** | 0.19* | 0.33** | 1.45** | 0.74** |  |

SUPPLEMENTARY TABLE S18. Population pairwise estimates of Slatkin’s linearized *F_ST_* (below diagonal) and $\phi$*_ST_* (above diagonal) based on variation in the *Bmal1* in band-rumped storm-petrels. Populations are arranged from north to south. Az = Azores; Berl = Berlengas; SH = St. Helena; CV = Cape Verde; Des = Desertas; AI = Ascension Island; Gal = Galapagos; Sel = Selvagem; (S) = summer-breeding; (W) = winter-breeding. All p-values were corrected using the FDR method. Statistical significance at p=0.05 and p=0.001 is indicated by * and **, respectively.

|  | **Japan** | **Hawaii** | **Gal (S)** | **Gal (W)** | **Berl** | **Az (S)** | **Az (W)** | **Des (S)** | **Des (W)** | **Sel (S)** | **Sel (W)** | **CV (S)** | **CV (W)** | **AI (S)** | **SH (W)** |
| --- | --- | --- | --- | --- | --- | --- | --- | --- | --- | --- | --- | --- | --- | --- | --- |
| **Japan** |  | 0.00 | 0.00 | 0.00 | 0.00 | 0.00 | 0.00 | 0.00 | 0.00 | 0.00 | 0.00 | 0.00 | 0.00 | 0.67* | 0.78* |
| **Hawaii** | 0.05 |  | 0.00 | 0.00 | 0.00 | 0.00 | 0.00 | 0.00 | 0.00 | 0.00 | 0.00 | 0.00 | 0.00 | 0.63 | 0.75* |
| **Gal (S)** | 0.05 | 0.03 |  | 0.00 | 0.00 | 0.00 | 0.00 | 0.00 | 0.00 | 0.00 | 0.00 | 0.00 | 0.00 | 0.49 | 0.66 |
| **Gal (W)** | 0.06 | 0.05 | 0.00 |  | 0.00 | 0.00 | 0.00 | 0.00 | 0.00 | 0.00 | 0.00 | 0.00 | 0.00 | 0.44 | 0.63 |
| **Berl** | 0.12 | 0.00 | 0.15 | 0.19 |  | 0.00 | 0.00 | 0.00 | 0.00 | 0.00 | 0.00 | 0.00 | 0.00 | 0.67* | 0.78* |
| **Az (S)** | 0.00 | 0.00 | 0.01 | 0.03 | 0.02 |  | 0.00 | 0.00 | 0.00 | 0.00 | 0.00 | 0.00 | 0.00 | 0.65* | 0.77* |
| **Az (W)** | 0.12 | 0.00 | 0.15 | 0.19 | 0.00 | 0.02 |  | 0.00 | 0.00 | 0.00 | 0.00 | 0.00 | 0.00 | 0.67* | 0.78* |
| **Des (S)** | 0.12 | 0.00 | 0.15 | 0.19 | 0.00 | 0.02 | 0.00 |  | 0.00 | 0.00 | 0.00 | 0.00 | 0.00 | 0.67* | 0.78* |
| **Des (W)** | 0.11 | 0.00 | 0.14 | 0.17 | 0.00 | 0.01 | 0.00 | 0.00 |  | 0.00 | 0.00 | 0.00 | 0.00 | 0.65* | 0.77* |
| **Sel (S)** | 0.13 | 0.00 | 0.17 | 0.20 | 0.00 | 0.02 | 0.00 | 0.00 | 0.00 |  | 0.00 | 0.00 | 0.00 | 0.68* | 0.79* |
| **Sel (W)** | 0.12 | 0.00 | 0.15 | 0.19 | 0.00 | 0.02 | 0.00 | 0.00 | 0.00 | 0.00 |  | 0.00 | 0.00 | 0.67* | 0.78* |
| **CV (S)** | 0.15 | 0.01 | 0.19 | 0.23 | 0.00 | 0.03 | 0.00 | 0.00 | 0.00 | 0.00 | 0.00 |  | 0.00 | 0.70* | 0.80* |
| **CV (W)** | 0.11 | 0.00 | 0.14 | 0.17 | 0.00 | 0.01 | 0.00 | 0.00 | 0.00 | 0.00 | 0.00 | 0.00 |  | 0.65* | 0.77* |
| **AI (S)** | 0.31* | 0.42* | 0.13 | 0.10 | 0.64* | 0.35* | 0.64* | 0.64* | 0.60* | 0.68** | 0.64* | 0.75** | 0.60* |  | -0.20 |
| **SH (W)** | 0.39* | 0.56** | 0.27* | 0.24* | 0.76** | 0.48** | 0.76** | 0.76** | 0.72** | 0.81** | 0.76** | 0.90** | 0.72** | 0.01 |  |

SUPPLEMENTARY TABLE 19. Population pairwise estimates of Slatkin’s linearized *F_ST_* (below diagonal) and $\phi$*_ST_* (above diagonal) based on variation in the *Bmal1* in Leach’s storm-petrels. Populations are arranged from north to south. Guad = Guadalupe; (S) = summer-breeding; (W) = winter-breeding. All p-values were corrected using the FDR method. Statistical significance at p=0.05 and p=0.001 is indicated by * and **, respectively.

|  | **Aiktak** | **Aleutians** | **BC** | **Japan** | **Guad (S)** | **Guad (W)** | **San Benito** |
| --- | --- | --- | --- | --- | --- | --- | --- |
| **Aiktak** |  | -0.08 | 0.00 | 0.13 | 0.00 | -0.04 | 0.23 |
| **Aleutians** | 0.00 |  | -0.49 | -0.32 | -0.19 | 0.06 | -0.25 |
| **BC** | 0.00 | 0.00 |  | 0.00 | 0.00 | 0.01 | 0.14 |
| **Japan** | 0.02 | 0.00 | 0.00 |  | 0.09 | 0.14 | -0.01 |
| **Guad (S)** | 0.00 | 0.00 | 0.00 | 0.00 |  | -0.01 | 0.20 |
| **Guad (W)** | 0.00 | 0.01 | 0.02 | 0.03 | 0.00 |  | 0.21 |
| **San Benito** | 0.15 | 0.05 | 0.07 | 0.04 | 0.06 | 0.10 |  |

SUPPLEMENTARY TABLE S20. Population pairwise estimates of Slatkin’s linearized *F_ST_* (below diagonal) and $\phi$*_ST_* (above diagonal) based on variation in *Bmal1* in Leach’s storm-petrels. Populations are arranged from north to south. SB = San Benito; Guad = Guadalupe; (S) = summer-breeding; (W) = winter-breeding. All p-values were corrected using the FDR method. No values were statistically significant.

|  | **Semidi** | **Aiktak** | **Buldir** | **Thomas** | **Storm** | **Cleland** | **Japan** | **Guad (S)** | **Guad (W)** | **SB** |
| --- | --- | --- | --- | --- | --- | --- | --- | --- | --- | --- |
| **Semidi** |  | -0.11 | -0.11 | -0.11 | -0.48 | -1.02 | -0.37 | -0.29 | 0.04 | -0.23 |
| **Aiktak** | 0.00 |  | -0.11 | 0.00 | 0.00 | 0.00 | 0.13 | 0.00 | -0.04 | 0.23 |
| **Buldir** | 0.00 | 0.01 |  | -0.11 | -0.48 | -1.02 | -0.62 | -0.29 | 0.07 | -0.44 |
| **Thomas** | 0.00 | 0.00 | 0.01 |  | 0.00 | 0.00 | 0.13 | 0.00 | -0.04 | 0.23 |
| **Storm** | 0.00 | 0.01 | 0.00 | 0.01 |  | 0.00 | -0.06 | 0.00 | -0.04 | 0.02 |
| **Cleland** | 0.00 | 0.05 | 0.00 | 0.05 | 0.00 |  | -0.27 | 0.00 | -0.04 | -0.22 |
| **Japan** | 0.00 | 0.02 | 0.00 | 0.02 | 0.00 | 0.00 |  | 0.09 | 0.14 | -0.01 |
| **Guad (S)** | 0.00 | 0.00 | 0.00 | 0.00 | 0.00 | 0.00 | 0.00 |  | -0.01 | 0.20 |
| **Guad (W)** | 0.00 | 0.00 | 0.00 | 0.00 | 0.00 | 0.05 | 0.03 | 0.00 |  | 0.21 |
| **SB** | 0.06 | 0.15 | 0.01 | 0.15 | 0.01 | 0.02 | 0.04 | 0.06 | 0.10 |  |

SUPPLEMENTARY TABLE S21. Population pairwise estimates of Slatkin’s linearized *F_ST_* (below diagonal) and $\phi$*_ST_* (above diagonal) based on variation in *Cry2* in band-rumped storm-petrels. Populations are arranged from north to south. Abbreviations are as follows: N = North; S = South; (S) = summer-breeding. All p-values were corrected using the FDR method. Statistical significance at p=0.05 and p=0.001 is indicated by the * and **, respectively.

|  | **Japan** | **Hawaii** | **Galapagos** | **Azores (S)** | **N Atlantic** | **Cape Verde** | **S Atlantic** |
| --- | --- | --- | --- | --- | --- | --- | --- |
| **Japan** |  | -0.14 | 0.55 | 0.03 | -0.49 | 0.46* | -0.47 |
| **Hawaii** | 0.00 |  | -0.02 | -0.11 | 0.61* | 0.41* | 0.21 |
| **Galapagos** | 0.08 | 0.00 |  | 0.06 | 0.82** | 0.41* | 0.60* |
| **Azores (S)** | 0.00 | 0.00 | 0.00 |  | 0.64* | 0.41* | 0.27 |
| **N Atlantic** | 0.18* | 0.51** | 0.76** | 0.47** |  | 0.81** | 0.11 |
| **Cape Verde** | 0.55** | 0.39** | 0.26** | 0.37** | 2.15** |  | 0.60** |
| **S Atlantic** | 0.00 | 0.10 | 0.24* | 0.09 | 0.04 | 0.89** |  |

SUPPLEMENTARY TABLE S22. Population pairwise estimates of Slatkin’s linearized *F_ST_* (below diagonal) and $\phi$*_ST_* (above diagonal) based on variation in the *Cry2* coding sequence in band-rumped storm-petrels. Since the *F_ST_* values are transformed using Slatkin’s linearization method, the values can be greater than 1. Populations are arranged from north to south. Az = Azores; Berl = Berlengas; SH = St. Helena; CV = Cape Verde; Des = Desertas; AI = Ascension Island; Gal = Galapagos; Sel = Selvagem; (S) = summer-breeding; (W) = winter-breeding. All p-values were corrected using the FDR method. Statistical significance at p=0.05 and p=0.001 is indicated by * and **, respectively.

|  | **Japan** | **Hawaii** | **Gal (S)** | **Gal (W)** | **Berl** | **Az (S)** | **Az (W)** | **Des (S)** | **Des (W)** | **Sel (S)** | **Sel (W)** | **CV (S)** | **CV (W)** | **AI (S)** | **SH (W)** |
| --- | --- | --- | --- | --- | --- | --- | --- | --- | --- | --- | --- | --- | --- | --- | --- |
| **Japan** |  | -0.14 | 0.74* | 0.48 | 0.00 | 0.03 | 0.00 | -0.94 | 0.00 | 0.00 | 0.00 | 0.40* | 0.75* | 0.00 | -0.69 |
| **Hawaii** | 0.00 |  | 0.24 | -0.57 | 0.48* | -0.11 | 0.48* | 0.14 | 0.31 | 0.39 | 0.36 | 0.36* | 0.61* | 0.27 | 0.05 |
| **Gal (S)** | 0.19 | 0.05 |  | 0.29 | 0.89** | 0.28 | 0.89* | 0.65* | 0.85* | 0.87* | 0.86* | 0.28 | 0.51* | 0.84* | 0.60 |
| **Gal (W)** | 0.00 | 0.00 | 0.03 |  | 0.78* | -0.38 | 0.78* | -0.11 | 0.71 | 0.74* | 0.72* | 0.32 | 0.65* | 0.68 | -0.19 |
| **Berl** | 0.33* | 0.58* | 1.22** | 0.60* |  | 0.55* | 0.00 | 0.11 | 0.00 | 0.00 | 0.00 | 0.55* | 0.85* | 0.00 | 0.27 |
| **Az (S)** | 0.00 | 0.00 | 0.05 | 0.00 | 0.49* |  | 0.55* | 0.17 | 0.40 | 0.47 | 0.44 | 0.37* | 0.64* | 0.36 | 0.08 |
| **Az (W)** | 0.33* | 0.58* | 1.22** | 0.60* | 0.00 | 0.49* |  | 0.11 | 0.00 | 0.00 | 0.00 | 0.55** | 0.85** | 0.00 | 0.27 |
| **Des (S)** | 0.00 | 0.12 | 0.47* | 0.14 | 0.12 | 0.11 | 0.12 |  | -0.22 | -0.08 | -0.11 | 0.46* | 0.75* | -0.27 | -0.11 |
| **Des (W)** | 0.01 | 0.15 | 0.53* | 0.17 | 0.10 | 0.14 | 0.10 | 0.00 |  | 0.00 | 0.00 | 0.51* | 0.83** | 0.00 | -0.01 |
| **Sel (S)** | 0.07 | 0.25 | 0.71** | 0.27* | 0.04 | 0.22 | 0.04 | 0.00 | 0.00 |  | 0.00 | 0.53* | 0.84** | 0.00 | 0.11 |
| **Sel (W)** | 0.05 | 0.22 | 0.64* | 0.24 | 0.06 | 0.20 | 0.06 | 0.00 | 0.00 | 0.00 |  | 0.51* | 0.83* | 0.00 | 0.08 |
| **CV (S)** | 0.50** | 0.38* | 0.24* | 0.32* | 1.71** | 0.36** | 1.71** | 0.82** | 0.89** | 1.10** | 1.02** |  | -0.11 | 0.49* | 0.42* |
| **CV (W)** | 0.78** | 0.48* | 0.18* | 0.41* | 3.46** | 0.46* | 3.46** | 1.39** | 1.52** | 1.95** | 1.80** | 0.05 |  | 0.81* | 0.72* |
| **AI (S)** | 0.00 | 0.12 | 0.47* | 0.14 | 0.12 | 0.11 | 0.12 | 0.00 | 0.00 | 0.00 | 0.00 | 0.82** | 1.39** |  | -0.06 |
| **SH (W)** | 0.00 | 0.03 | 0.29* | 0.04 | 0.23 | 0.03 | 0.23 | 0.00 | 0.00 | 0.02 | 0.00 | 0.60** | 0.98** | 0.00 |  |

SUPPLEMENTARY TABLE 23. Population pairwise estimates of Slatkin’s linearized *F_ST_* (below diagonal) and $\phi$*_ST_* (above diagonal) based on variation in *Cry2* in Leach’s storm-petrels. Populations are arranged from north to south. (S) = summer-breeding; (W) = winter-breeding. All p-values were corrected using the FDR method [37]. Statistical significance at p=0.05 and p=0.001 is indicated by * and **, respectively.

|  | **Aiktak** | **Aleutians** | **BC** | **Japan** | **Guadalupe (S)** | **Guadalupe (W)** | **San Benito** |
| --- | --- | --- | --- | --- | --- | --- | --- |
| **Aiktak** |  | 0.12* | 0.01 | 0.23 | -0.02* | 0.03** | 0.19 |
| **Aleutians** | 0.20 |  | 0.19* | 0.05* | 0.16* | 0.30* | 0.04* |
| **BC** | 0.00 | 0.24* |  | 0.27 | -0.04** | 0.00** | 0.24 |
| **Japan** | 0.00 | 0.42* | 0.00 |  | 0.25** | 0.37** | -0.01 |
| **Guadalupe (S)** | 0.33** | 0.05 | 0.36** | 0.58** |  | -0.03 | 0.21* |
| **Guadalupe (W)** | 0.27** | 0.04 | 0.32** | 0.48** | 0.00 |  | 0.33** |
| **San Benito** | 0.00 | 0.14 | 0.01 | 0.04 | 0.29** | 0.23* |  |

SUPPLEMENTARY TABLE S24. Population pairwise estimates of Slatkin’s linearized *F_ST_* (below diagonal) and $\phi$*_ST_* (above diagonal) based on variation in *Cry2* in Leach’s storm-petrels. Populations are arranged from north to south. SB = San Benito; Guad = Guadalupe; (S) = summer-breeding; (W) = winter-breeding. All p-values were corrected using the FDR method. Statistical significance at p=0.05 and p=0.001 is indicated by * and **, respectively.

|  | **Semidi** | **Aiktak** | **Buldir** | **Thomas** | **Storm** | **Cleland** | **Japan** | **Guad (S)** | **Guad (W)** | | **SB** |
| --- | --- | --- | --- | --- | --- | --- | --- | --- | --- | --- | --- |
| **Semidi** |  | 0.62* | -0.08 | 0.57* | 0.57* | 0.79* | 0.79* | 0.51* | 0.56* | 0.67* | |
| **Aiktak** | 0.38* |  | -4.14 | -0.13 | -0.16 | -0.22 | -0.07 | 0.61* | 0.68* | -0.48 | |
| **Buldir** | 0.05 | 0.00 |  | -4.14 | -4.60 | 0.00 | -3.30 | -0.22 | 0.18 | 0.00 | |
| **Thomas** | 0.20 | 0.00 | 0.00 |  | -0.26 | 0.46 | 0.08 | 0.49* | 0.59* | 0.23 | |
| **Storm** | 0.47* | 0.00 | 0.03 | 0.03 |  | -0.40 | -0.12 | 0.58* | 0.66* | -0.70 | |
| **Cleland** | 0.52* | 0.00 | 0.06 | 0.05 | 0.00 |  | -0.15 | 0.75** | 0.77** | 0.00 | |
| **Japan** | 0.71* | 0.00 | 0.12 | 0.09 | 0.00 | 0.00 |  | 0.73** | 0.77** | -0.33 | |
| **Guad (S)** | 0.12 | 0.33* | 0.00 | 0.12 | 0.40* | 0.44** | 0.58** |  | 0.06 | 0.66* | |
| **Guad (W)** | 0.09 | 0.27* | 0.00 | 0.11* | 0.33** | 0.36** | 0.48** | 0.00 |  | 0.70** | |
| **SB** | 0.25* | 0.00 | 0.00 | 0.02 | 0.00 | 0.00 | 0.04 | 0.29** | 0.23* |  | |

SUPPLEMENTARY TABLE S25. Pairwise estimates of Weir and Cockerham F_ST_ values comparing genetic differences between the West, Central, and East San Benito Island colonies of Leach’s storm-petrels based on neutral loci.

|  | **West Benito** | **Central Benito** | **East Benito** |
| --- | --- | --- | --- |
| **West Benito** |  | 0.00 | 0.00 |
| **Central Benito** | 0.00 |  | 0.00 |
| **East Benito** | 0.00 | 0.00 |  |


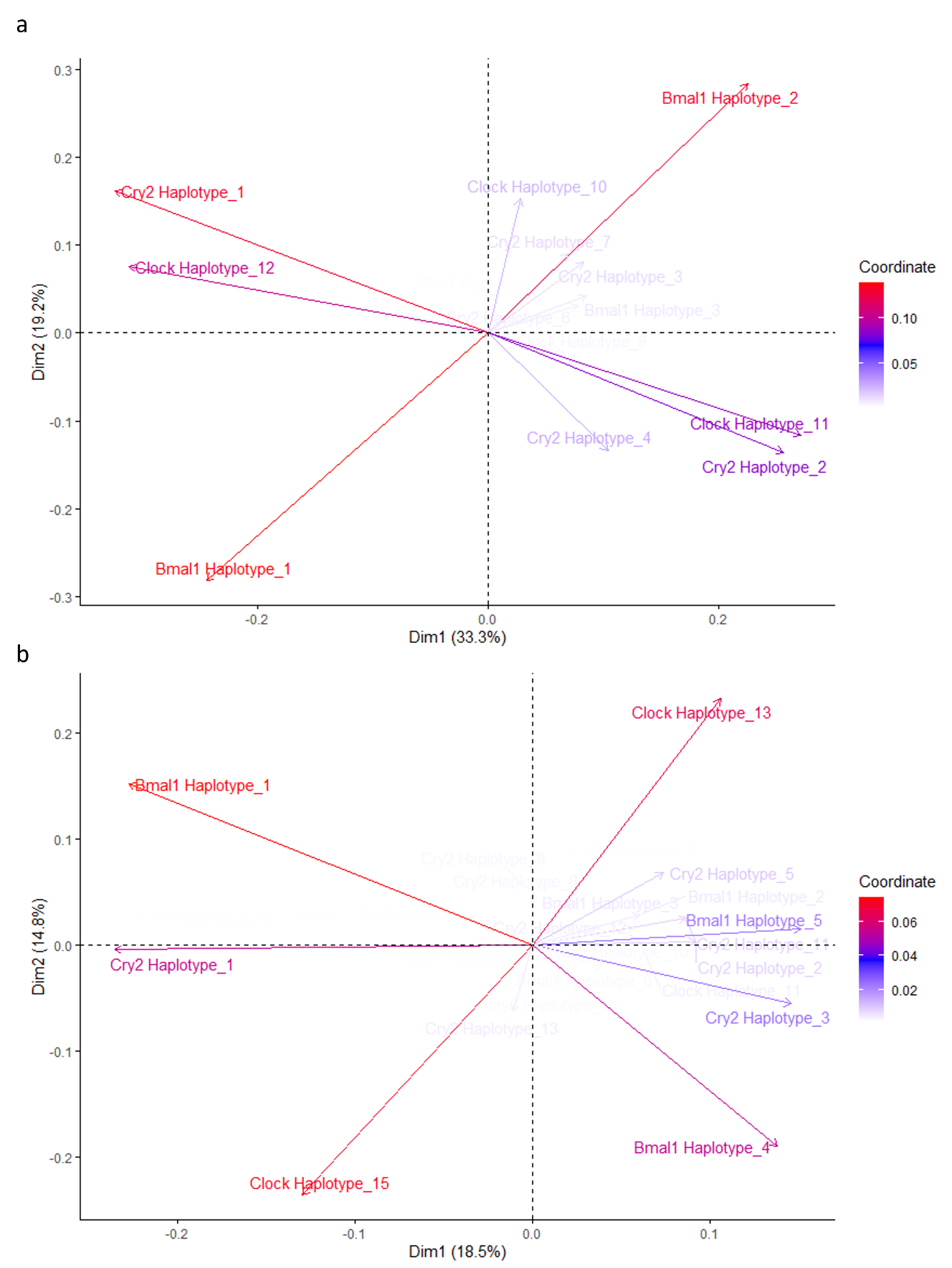
SUPPLEMENTARY FIGURE 1. Principal Components Analysis (PCA) depicting how the different alleles of *Clock*, *Bmal1,* and *Cry2* vary together in a) band-rumped storm-petrels and b) Leach’s storm-petrels.


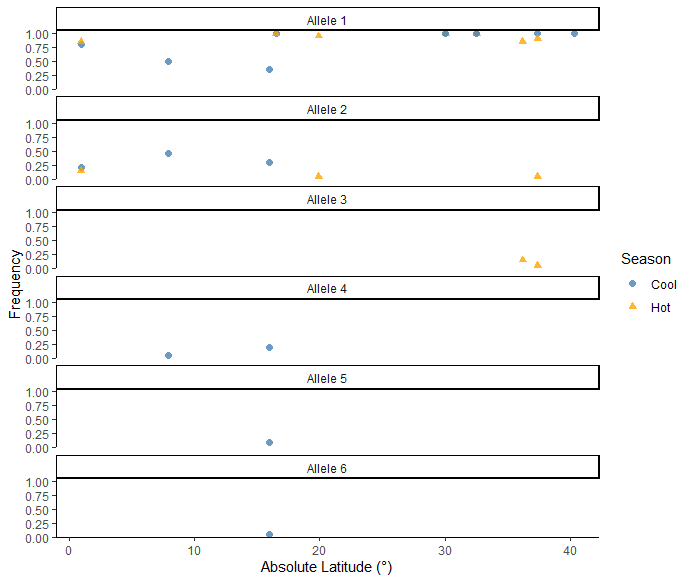


SUPPLEMENTARY FIGURE 2. *Bmal1* allele frequencies by absolute latitude for band-rumped storm-petrels.


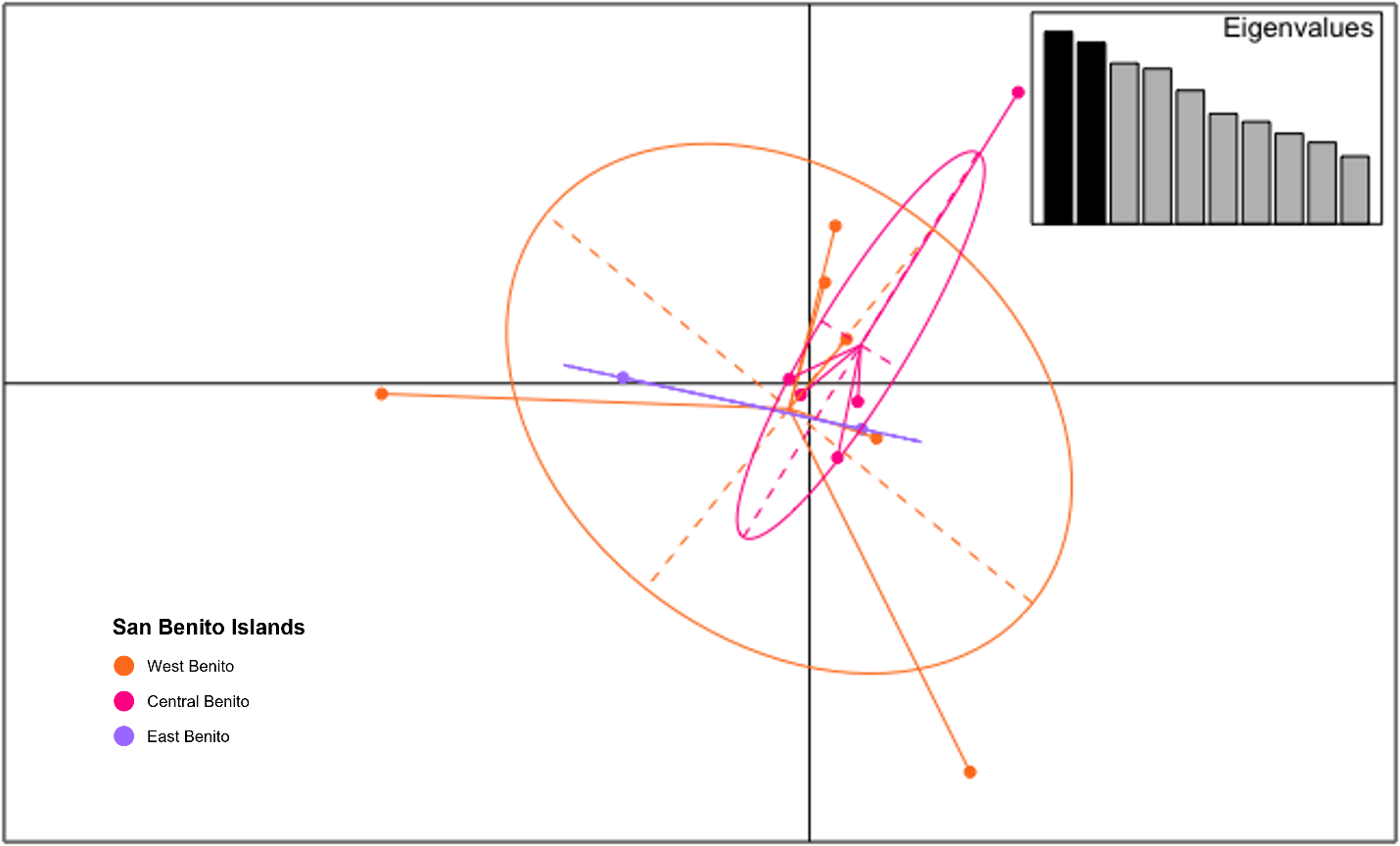


SUPPLEMENTARY FIGURE 3. Principal Components Analysis (PCA) depicting genetic variation across the West, Central, and East San Benito Island colonies of Leach’s storm-petrels based on neutral loci.
